# ﻿New species and records of ascomycetes on cypress in Beijing, China

**DOI:** 10.3897/mycokeys.123.165848

**Published:** 2025-10-16

**Authors:** Zixian Bi, Yingying Wu, Shuji Li, Chengming Tian

**Affiliations:** 1 The Key Laboratory for Silviculture and Conservation of the Ministry of Education, Beijing Forestry University, Beijing 100083, China Beijing Forestry University Beijing China

**Keywords:** Ascomycota, cypress, morphology, multi-gene phylogenetic, taxonomy

## Abstract

Cypress, a collective term for plants belonging to the Cupressaceae family, is widely utilized in Chinese landscaping and holds considerable economic and medicinal importance. In recent years, dieback of branches and foliage in cypress has been widespread in the Ming Tombs area of Beijing, yet the associated ascomycetous fungi remain unidentified. During an investigation of the species of ascomycetes associated with three cypress species (*Juniperus chinensis*, *J. procumbens*, and *Platycladus orientalis*) in the Ming Tombs area of Beijing, 22 fungal strains were isolated from withered branches and diseased leaves to healthy strobili and mature cones. Based on integrated morphological and multi-gene phylogenetic analyses, these strains were identified as 13 fungal species belonging to 8 genera. Among these, two novel species—*Nigrospora platycladiensis***sp. nov.** and *Spegazzinia juniperi***sp. nov.**—and two new host records, *Aplosporella hesperidica* and *Nigrospora philosophiae-doctoris*, are reported herein. This study contributes to our understanding of the richness of ascomycetes on cypress.

## ﻿Introduction

*Nigrospora* was introduced by Zimmerman (1902) with *N. panici* designated as the type species (Wang et al. 2017). As an important genus of ascomycetous fungi, *Nigrospora* commonly exists as plant pathogens, endophytes, or saprophytes and is widely distributed across various plants, as well as in soil and air (Wang et al. 2017; Raza et al. 2019). The defining characteristics of *Nigrospora* include its dark-pigmented conidia and conidiophores (Zimmerman 1902; Wang et al. 2024a; Zou et al. 2024). Initially, species identification within *Nigrospora* relied on morphological comparisons of conidial size and structure (Mason 1927; Zou et al. 2024). However, interspecific morphological distinctions in this genus are often subtle—thus taxonomic identification necessitates an integrative approach combining morphological examination with phylogenetic evidence (Hao et al. 2020; Wang et al. 2017). Since then, numerous novel *Nigrospora* species have been discovered (Raza et al. 2019; Tian et al. 2020; Liu et al. 2024; Wang et al. 2024a).

*Spegazzinia* was established by Saccardo (1880) with *S. ornata* (now treated as a synonym of *S. tessarthra*) designated as its type species. *Spegazzinia* exhibits a remarkably broad geographic distribution, predominantly existing as endophytes within host organisms or as saprophytes colonizing decaying plant debris (Hashemlou et al. 2023; Dai et al. 2025). Hyde et al. (1998) initially classified this genus within Sordariomycetes (Apiosporaceae) based on its morphological characteristic of basauxic conidiogenesis. Subsequently, Tanaka et al. (2015) reclassified the genus into Dothideomycetes (Didymosphaeriaceae) based on phylogenetic evidence derived from three multi-gene loci. In recent years, many new species of *Spegazzinia* have been described (Jayasiri et al. 2019; Samarakoon et al. 2020; Hashemlou et al. 2023; Zhang et al. 2024a; Dai et al. 2025). The genus is characterized by basauxic conidiogenesis, producing brown to dark brown conidia that may exhibit spine-like appendages. Notably, it develops two distinct conidial morphotypes: stellate α-conidia and cloverleaf-shaped β-conidia (Suwannarach et al. 2021; Hashemlou et al. 2023; Zhang et al. 2024a).

Cypress belongs to the family Cupressaceae, a general term encompassing various cypress species, including *Juniperus chinensis*, *Juniperus procumbens*, and *Platycladus orientalis* (Su and Zou 2023). Cupressaceae plants are widely distributed worldwide, comprising approximately 22 genera and nearly 150 species (Shang et al. 2013). In China, there are about 8 genera and over 30 species of cypress, with 1 additional genus introduced through cultivation (Shang et al. 2013; Lin et al. 2021). Cypresses are extensively planted across all districts of Beijing, primarily featuring *P. orientalis* and *J. chinensis*, with *P. orientalis* designated as Beijing’s official city tree (Duan et al. 2024). Renowned for their elegant forms, exceptional environmental adaptability, and unique chemical composition, cypress trees play crucial roles in China’s urban landscaping, economic development, ecological conservation, and medical applications (Gong et al. 2020; Duan et al. 2024).

The phylum Ascomycota, comprising the largest number of species within the fungal kingdom, is widely distributed across various host plants (Hyde et al. 2019). With the rise of molecular systematics, ascomycete groups related to cypresses have been further explored, and many new species have been successively reported (Pan et al. 2021; Peng et al. 2023; Jiao et al. 2024; Lin et al. 2024; Jiao et al. 2025b; Zhou et al. 2025). Zhu (1922) first discovered leaf blight disease of *P. orientalis* caused by *Alternaria pruni* in China. Later, Liu et al. (1997) observed a large-scale outbreak of shoot blight disease on *J. chinensis* in the Dalian region. They identified both the pathogen and its infection patterns, confirming that the disease was caused by a fungal species belonging to *Coniothyrium* sp. Li et al. (2010) reported for the first time in China that *Neofusicoccum parvum* caused cypress dieback disease. Subsequently, Mohammadi et al. (2014) isolated *N. parvum* from discolored, necrotic, cankered, and wilted wood tissues of cypress trees during their investigation of cypress decline disease in southeastern Iran. Cypress canker disease is a severe fungal disease worldwide (Boesewinkel 1983; Graniti 1986, 1998; Danti and Della Rocca 2017). This disease was first reported as an epidemic on Monterey cypress in California (Wagener 1928; Danti and Della Rocca 2017). Initially prevalent across North America, South America, Africa, Australia, and New Zealand, it subsequently spread eastward to other regions (Wagener 1928; Urbasch 1993). According to international literature, *Seiridium cardinale*, *S. cupressi*, and *S. unicorne* are among the most destructive pathogenic fungi affecting Cupressaceae plants and are recognized as the primary causative agents of cypress canker epidemics (Boesewinkel 1983; Graniti 1986, 1998; Danti and Della Rocca 2017; Bonthond et al. 2018). Guo (2023) made the first documented discovery of cypress canker disease caused by *S. unicorne* in China. In recent years, studies have demonstrated that *Alternaria* spp., *Nothophoma* spp., *Bipolaris setariae*, and *B. sorokiniana* are pathogenic fungi capable of causing leaf blight in ancient *P. orientalis* trees in the Beijing region (Jiao et al. 2024, 2025a, 2025b). While studies have documented ascomycete communities associated with cypress trees, research efforts have primarily concentrated on pathogenic groups, with cypress pathogens receiving significant attention from researchers both in China and internationally.

In addition to pathogenic species, cypress-associated ascomycetes include diverse endophytic fungi whose secondary metabolites exhibit insecticidal, antimicrobial, and antitumor bioactivities (Rawat et al. 2010; Ismail et al. 2013). For instance, Zhang et al. (2014) isolated *Chaetomium globosum* from *P. orientalis* foliage, with subsequent bioassays demonstrating its metabolites’ potent inhibitory effects against pathogenic bacteria. However, current research on endophytic fungi of cypresses in China remains limited, with most studies primarily focusing on their antibacterial and antifungal activities (Wang et al. 2010; Zhang et al. 2014).

In recent years, the phenomenon of branch and leaf withering in cypress has become increasingly common in the Ming Tombs area of Beijing. However, the diversity of Ascomycota fungi associated with these trees remains unclear. Most existing studies on cypress-related fungi focus primarily on pathogenic species, while research on endophytic and saprophytic fungi is relatively scarce. In this study, we collected 22 strains of Ascomycota fungi from various parts of three cypress species (*J. chinensis*, *J. procumbens*, and *P. orientalis*), including withered branches, diseased leaves, healthy strobili, and mature cones, in the Ming Tombs area of Beijing. Through morphological and molecular phylogenetic analyses, detailed identification of known species and potential new species was conducted. This research not only provides fundamental data for future studies on the diversity of cypress-associated Ascomycota but also expands the known diversity of these fungi.

## ﻿Materials and methods

### ﻿Sample collection and fungal isolation

**Figure 1.**
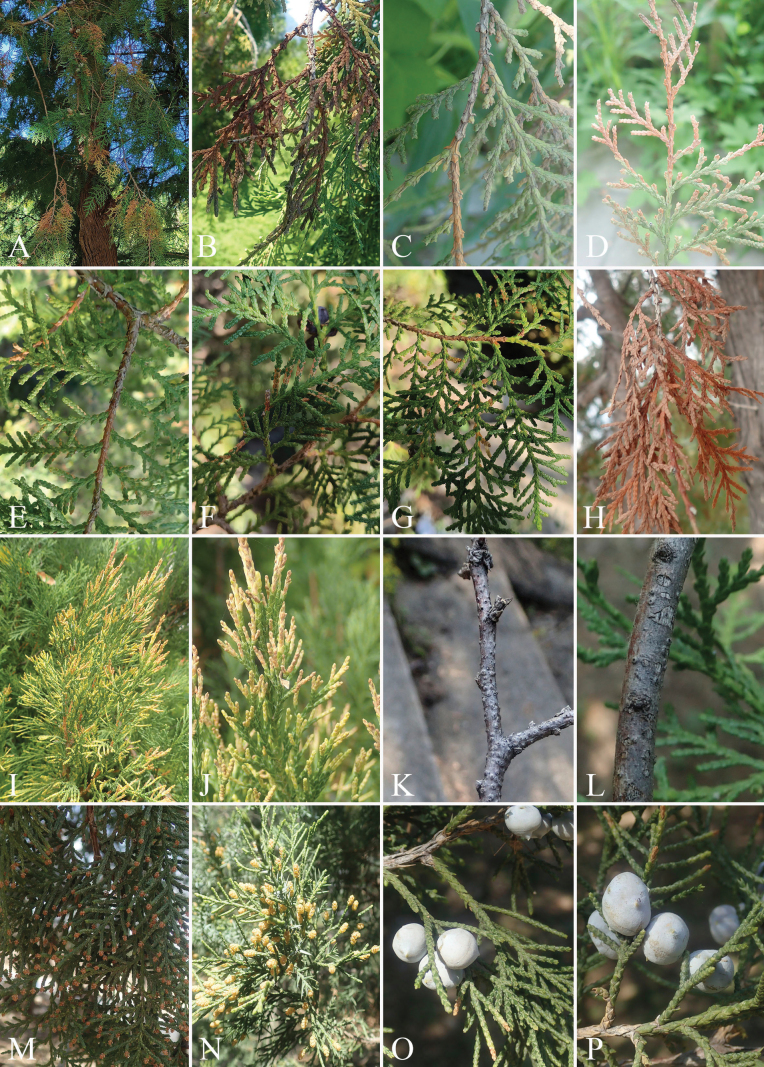
Collection type of Ascomycota specimens related to cypress in the Ming Tombs area, Beijing. A–H. Diseased leaves of *Platycladus orientalis*; I, J. Dieback twigs of *Juniperus procumbens*; K, L. Dead branches on *Platycladus orientalis*; M. Healthy strobili of *Platycladus orientalis*; N. Healthy strobili of *Juniperus chinensis*; O, P. Healthy cones of *Juniperus chinensis*.

During a diversity survey of ascomycetous fungi associated with cypress in the Ming Tombs area of Beijing, specimens were collected from 3 cypress species (*Juniperus chinensis*, *J. procumbens*, and *Platycladus orientalis*) at multiple sites, including Dingling Tomb, Changling Tomb, Longshan Sub-Farm of the Ming Tombs, Beijing Mangshan National Forest Park, Beijing Dayu Mountain Scenic Area, and the Ming Tombs Reservoir. A total of 18 specimens were collected: *P. orientalis* (15 specimens), *J. chinensis* (2 specimens), and *J. procumbens* (1 specimen), including diseased branches, infected leaves, as well as healthy strobili and mature cones (Fig. 1). The fungal species isolated from various tissues of different host plants in this study are detailed in Suppl. material 1. All specimens were transported to the laboratory in sealed plastic bags for further analysis. Before separation, the surface of each specimen was rinsed with tap water to remove dust and then placed in a laminar flow cabinet. Using a sterilized blade, tissue blocks (approximately 0.5 × 0.5 cm) were cut from the disease–health junction, infection sites, and surfaces of healthy strobili and mature cones. The samples were sequentially treated by soaking in 75% ethanol for 30 seconds, followed by 5% NaClO for 1 minute, and then rinsed three times with sterile water. Surface moisture was absorbed with sterilized filter paper, and the samples were transferred to potato dextrose agar plates (PDA: prepared with 200 g potatoes, 20 g glucose, and 20 g agar; 1 liter of sterile water) and incubated at 25 °C for 2–5 days. After colonies appeared, a sterilized inoculation needle was used to pick hyphae from the edge of the colony and transfer them to PDA medium to obtain pure cultures (Yuan et al. 2024). For specimens with clearly visible fruiting bodies, spore masses were aseptically transferred to the surface of PDA medium and cultured under dark conditions at 25 °C (Wang et al. 2024b). After colony formation, they were transferred to fresh PDA plates for further cultivation. The specimens used in this study are deposited in the Museum of Beijing Forestry University (BJFC), and the fungal strains are preserved at the China Forestry Culture Collection Center (CFCC).

### ﻿Morphological observation

The cultures were inoculated onto PDA medium and incubated at 25 °C under dark conditions for 14–30 days, with regular documentation of colony morphological characteristics (Yuan et al. 2024). After sporulation structures developed, the morphological features of fruiting bodies were examined using a stereomicroscope (OLYMPUS SZX2-FOF, Tokyo, Japan). Microscopic characteristics, including asci, ascospores, conidia, and conidiophores, were observed under a Nikon Eclipse 80i compound microscope equipped with differential interference contrast (DIC) illumination. Photographic documentation was performed using Nikon Nis-Elements F4.30.01 software. For naturally developed fruiting bodies on host branches, transverse and longitudinal sections were prepared using sterile double-edged razor blades for microscopic examination and documentation. Morphometric analyses were performed on 15–30 pycnidia, asci, and conidiogenous cells, along with 30–50 spores.

### ﻿DNA extraction, PCR amplification, and sequencing

After approximately 10 days of culture, fungal DNA was extracted using a modified CTAB method (Doyle 1990). Based on genus-specific diagnostic characteristics documented in the literature, corresponding genetic markers were selected for PCR amplification. For *Aplosporella*, the internal transcribed spacer region rDNA (ITS) (primers; ITS1/ITS4) and the translation elongation factor 1-alpha (*tef1*) (primers; EF1-728F/EF1-986R) gene were amplified (Lin et al. 2023a). For *Achaetomium*, *Arcopilus*, and *Chaetomium*, the internal transcribed spacer region rDNA (ITS) (primers; ITS1/ITS4), large subunit ribosomal RNA (LSU) (primers; LR5/LROR), the partial beta-tubulin (*tub2*) (primers; T1/TUB4Rd) gene, and the RNA polymerase II second largest subunit (*rpb2*) (primers; fRPB2-5F/fRPB2-7cR) loci were amplified (Wang et al. 2022). For *Seiridium*, the internal transcribed spacer region rDNA (ITS) (primers; ITS1/ITS4), large subunit ribosomal RNA (LSU) (primers; LR5/LROR), the translation elongation factor 1-alpha (*tef1*) (primers; EF1-728F/EF2) gene, the partial beta-tubulin (*tub2*) (primers; T1/BT2b) gene, and the RNA polymerase II second largest subunit (*rpb2*) (primers; fRPB2-5F/fRPB2-7cR) loci were amplified (Dissanayake et al. 2021). For *Nigrospora*, the internal transcribed spacer region rDNA (ITS) (primers; ITS1/ITS4), the translation elongation factor 1-alpha (*tef1*) (primers; EF1-728F/EF2 or EF1-728F/EF1-986R) gene, and the partial beta-tubulin (*tub2*) (primers; BT2a/BT2b) gene were amplified (Wang et al. 2017; Liu et al. 2024). For *Neofusicoccum*, the internal transcribed spacer region rDNA (ITS) (primers; ITS1/ITS4), the translation elongation factor 1-alpha (*tef1*) (primers; EF1-728F/EF1-986R) gene, the partial beta-tubulin (*tub2*) (primers; BT2a/BT2b) gene, and the RNA polymerase II second largest subunit (*rpb2*) (primers; fRPB2-5F/fRPB2-7cR) loci were amplified (Si et al. 2023). For *Spegazzinia*, the internal transcribed spacer region rDNA (ITS) (primers; ITS1/ITS4), the translation elongation factor 1-alpha (*tef1*) (primers; EF1-983F/EF1-2218R) gene, the large subunit ribosomal RNA (LSU) (primers; LR5/LROR), and the small subunit rDNA (SSU) (primers; NS1/NS4) were amplified (Zhang et al. 2024a). The primer sequences (forward and reverse) and PCR conditions for each genus are provided in Suppl. material 2. PCR amplification was performed in a 20 μL reaction system containing 10 μL 2×ES Taq Mastermix (Dye), 7 μL double deionized water, 1 μL template DNA, and 1 μL of each primer. Amplification products were electrophoresed on 2% agarose gels (Wu et al. 2024) and subsequently sequenced by Tsingke Biotechnology Co., Ltd. (Beijing). The sequences newly obtained in this study have been submitted to GenBank, and the accession numbers have been obtained. The sequences obtained in this study are in Suppl. material 2.

### ﻿Phylogenetic analyses

The obtained sequences were first assembled using SeqMan v. 7.1.0 software. Subsequently, BLAST analysis was performed in the NCBI database (https://www.ncbi.nlm.nih.gov/) to retrieve reference sequences for relevant genera from previously published articles (Samarakoon et al. 2020; Dissanayake et al. 2021; Li et al. 2022; Xu et al. 2022; Condé et al. 2023; Lin et al. 2023a; Si et al. 2023; Qian et al. 2024; Wu et al. 2024; Zhang et al. 2024a, 2024b, 2024c; Zou et al. 2024; Dai et al. 2025). The acquired sequences are listed in Suppl. material 2. Sequence alignment was conducted using MAFFT v. 7 (https://mafft.cbrc.jp/alignment/server/) (Katoh and Standley 2013), and the sequences were aligned and edited using MEGA v. 6 software (Tamura et al. 2013). Phylogenetic trees based on multiple genes were constructed using Maximum Likelihood (ML) and Bayesian inference (BI) methods, implemented with PhyML v. 3.0 and MrBayes v. 3.1.2 software, respectively (Huelsenbeck and Ronquist 2001; Swofford 2003; Silvestro and Michalak 2012). The phylograms were visualized using FigTree v. 1.4.0 software and then edited using Adobe Illustrator CS v. 5. Finally, nodes with bootstrap support values ≥ 50% in the ML analyses, and posterior probabilities (PP) ≥ 0.90 in the BI analysis, were clearly labeled on each phylogram.

## ﻿Results

### ﻿Phylogenetic analyses

Phylogenetic analyses of *Nigrospora*

**Figure 2.**
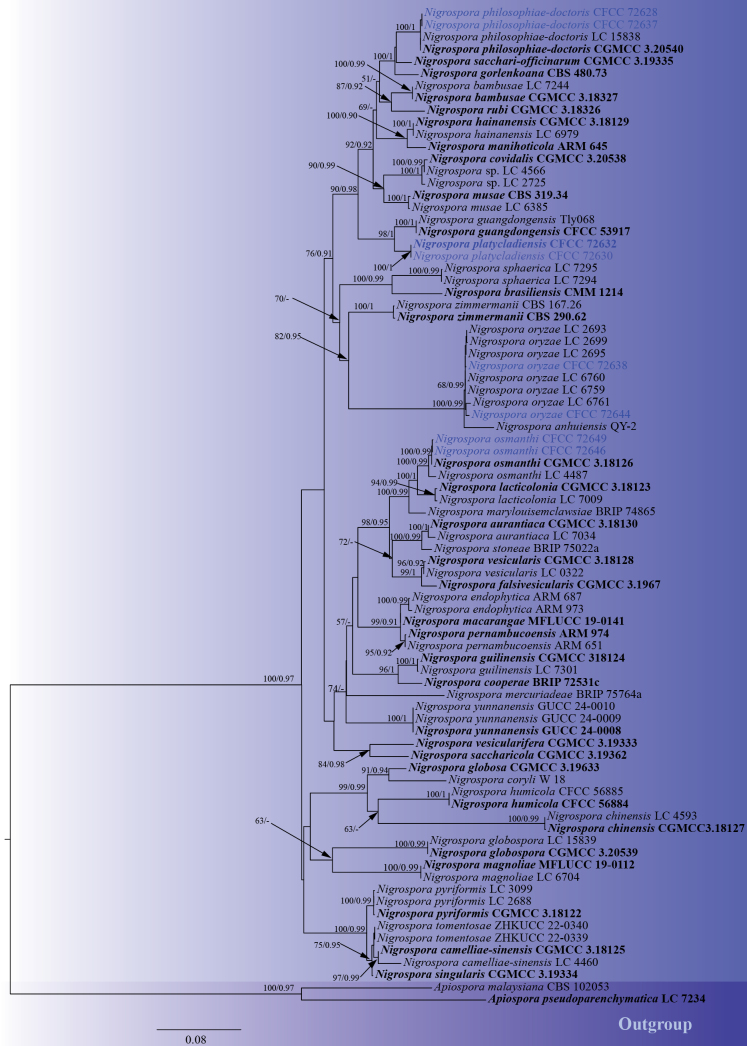
ML phylogenetic tree of *Nigrospora* based on combined ITS, *tef1-α*, and *tub2* sequence data. The tree is rooted with *Apiospora malaysiana* (CBS 102053) and *Apiospora pseudoparenchymatica* (LC 7234). Bootstrap support values from ML analysis (ML ≥ 50%) and Bayesian posterior probabilities (BI ≥ 0.90) are shown at the nodes. Strains obtained in this study are marked in blue; ex-type strains are indicated in bold black type.

In this study, eight *Nigrospora* strains were isolated from *Juniperus chinensis*, *Juniperus procumbens*, and *Platycladus orientalis*. A multigene phylogenetic tree of the genus *Nigrospora* was constructed using concatenated sequences of the ITS, *tef1-α*, and *tub2* genes, with *Apiospora malaysiana* (CBS 102053) and *Apiospora pseudoparenchymatica* (LC 7234) designated as outgroups (Zou et al. 2024). The phylogenetic tree revealed that the eight strains formed four distinct clades: the strains CFCC 72628 and CFCC 72637 clustered with *Nigrospora philosophiae-doctoris* (CGMCC 3.20540) with strong support values of 100/1 (ML/BI); the strains CFCC 72638 and CFCC 72644 clustered with *Nigrospora oryzae* with strong support values of 100/0.99 (ML/BI); the strains CFCC 72646 and CFCC 72649 clustered with *Nigrospora osmanthi* (CGMCC 3.18126) with strong support values of 100/0.99 (ML/BI); and the strains CFCC 72630 and CFCC 72632 formed an independent branch, with strong support values of 100/1 (ML/BI), representing a novel species (Fig. 2). The phylogenetic tree contained a total of 1615 characters, comprising 935 constant characters, 129 variable characters, and 551 parsimony-informative characters. In the ML analysis based on the combined gene dataset, the matrix possessed 866 distinct alignment patterns. Estimated base frequencies are as follows: A = 0.214835, C = 0.305355, G = 0.239540, T = 0.240270, AC = 0.948986, AG = 2.819556, AT = 0.889264, CG = 0.897776, CT = 4.263318, GT = 1.000000, and gamma distribution shape parameter: α = 0.233074. The phylogenetic trees constructed using ML and BI exhibited identical topology.

#### ﻿Phylogenetic analyses of *Spegazzinia*

**Figure 3.**
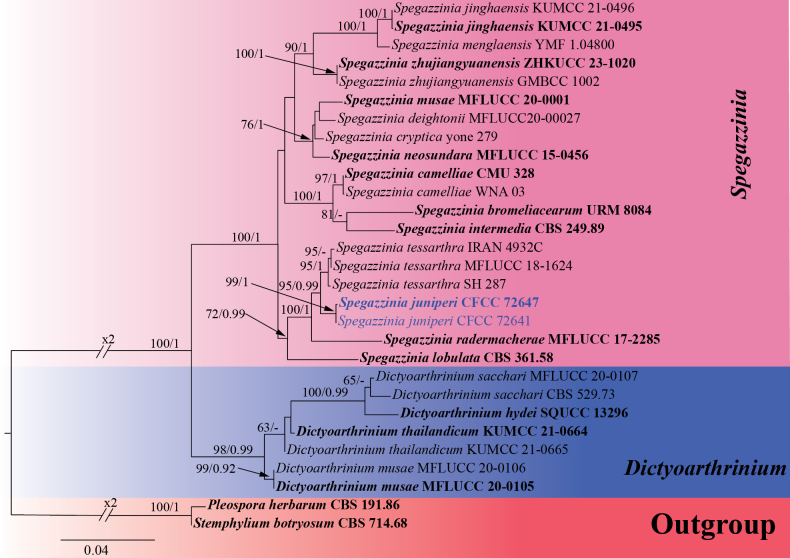
ML phylogenetic tree of *Spegazzinia* and its closely related genera based on combined ITS, LSU, SSU, and *tef1-α* sequence data. The tree is rooted with *Pleospora herbarum* (CBS 191.86) and *Stemphylium botryosum* (CBS 714.68). Bootstrap support values from ML analysis (ML ≥ 50%) and Bayesian posterior probabilities (BI ≥ 0.90) are shown at the nodes. Strains obtained in this study are marked in blue; ex-type strains are indicated in bold black type.

In this study, two *Spegazzinia* strains were isolated from healthy cones of *Juniperus chinensis*. A multigene phylogenetic tree of the *Spegazzinia* and its closely related genera was constructed using concatenated sequences of the ITS, LSU, SSU, and *tef1-α* genes, with reference sequences derived from previously published studies (Samarakoon et al. 2020; Hashemlou et al. 2023; Zhang et al. 2024a; Dai et al. 2025). The complete phylogenetic tree of Didymosphaeriaceae is provided in Suppl. material 3. *Pleospora herbarum* (CBS 191.86) and *Stemphylium botryosum* (CBS 714.68) were selected as the outgroup (Samarakoon et al. 2020). The phylogenetic tree revealed that the two isolated strains, CFCC 72641 and CFCC 72647, formed a distinct clade with strong support values of 99/1 (ML/BI) (Fig. 3). This clade did not cluster with any known species, indicating their status as a novel species. The phylogenetic tree contained a total of 3455 characters, comprising 2733 constant sites, 182 variable sites, and 540 parsimony-informative characters. In the ML analysis based on the combined gene dataset, the matrix possessed 829 distinct alignment patterns. Estimated base frequencies are as follows: A = 0.234832, C = 0.261337, G = 0.275448, T = 0.228383, AC = 1.194130, AG = 2.107716, AT = 1.188755, CG = 1.166085, CT = 5.918954, GT = 1.000000, and gamma distribution shape parameter: α = 0.161473. The phylogenetic trees constructed using ML and BI exhibited identical topology.

#### ﻿Phylogenetic analyses of *Aplosporella*

**Figure 4.**
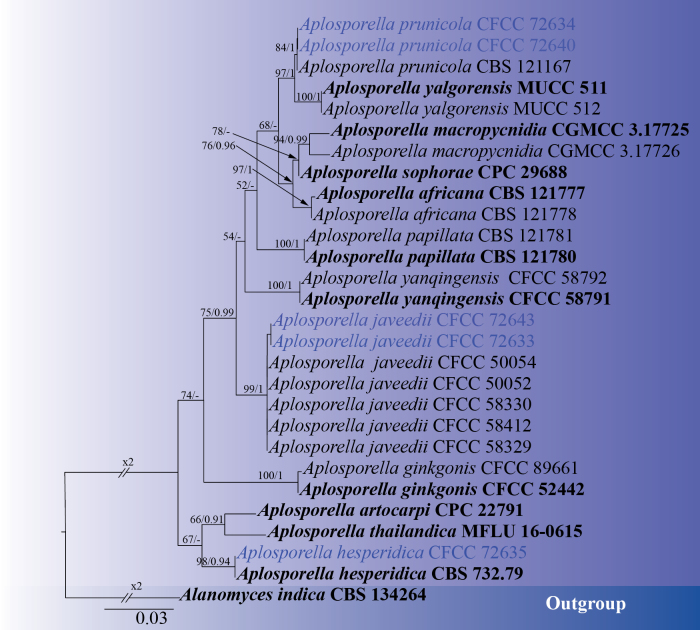
ML phylogenetic tree of *Aplosporella* based on combined ITS and *tef1-α* sequence data. The tree is rooted with *Alanomyces indica* (CBS 134264). Bootstrap support values from ML analysis (ML ≥ 50%) and Bayesian posterior probabilities (BI ≥ 0.90) are shown at the nodes. Strains obtained in this study are marked in blue; ex-type strains are indicated in bold black type.

In this study, five *Aplosporella* strains were isolated from *Platycladus orientalis* in the Ming Tombs area of Beijing. A multi-gene phylogenetic tree of the genus *Aplosporella* was constructed based on the combined ITS and *tef1-α* gene regions, with *Alanomyces indica* (CBS 134264) selected as the outgroup (Lin et al. 2023a; Wu et al. 2024). The phylogenetic tree revealed that these five strains clustered into three distinct clades: strain CFCC 72635 clustered with *Aplosporella hesperidica* (CBS 732.79) with support values of 98/0.94 (ML/BI); strains CFCC 72633 and CFCC 72643 grouped with *Aplosporella javeedii* with support values of 99/1 (ML/BI); and strains CFCC 72634 and CFCC 72640 grouped with *Aplosporella prunicola* (CBS 121167) with support values of 84/1 (ML/BI) (Fig. 4). The phylogenetic tree contained a total of 831 characters, comprising 668 constant characters, 73 variable characters, and 90 parsimony-informative characters. In the ML analysis based on the combined gene dataset, the matrix possessed 207 distinct alignment patterns. Estimated base frequencies are as follows: A = 0.211875, C = 0.267409, G = 0.257202, T = 0.263513, AC = 2.898147, AG = 3.745261, AT = 1.882649, CG = 2.351749, CT = 6.230615, GT = 1.000000, and gamma distribution shape parameter: α = 0.214122. The phylogenetic trees constructed using ML and BI exhibited identical topology.

#### ﻿Phylogenetic analyses of *Neofusicoccum*

**Figure 5.**
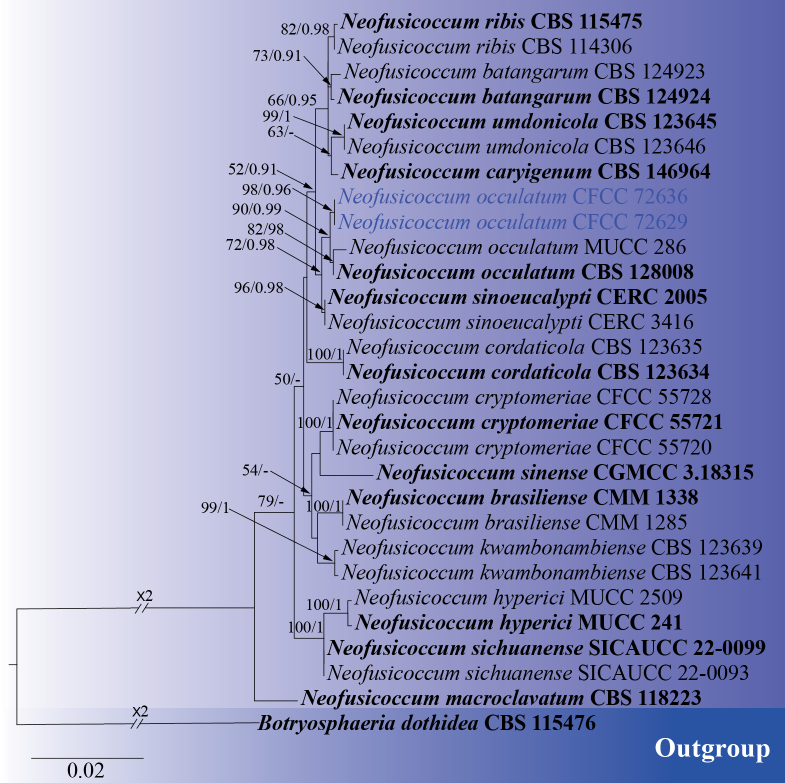
ML phylogenetic tree of *Neofusicoccum* isolates based on combined ITS, *tef1-α*, *tub2*, and *rpb2* sequence data. The tree is rooted with *Botryosphaeria dothidea* (CBS 115476). Bootstrap support values from ML analysis (ML ≥ 50%) and Bayesian posterior probabilities (BI ≥ 0.90) are shown at the nodes. Strains obtained in this study are marked in blue; ex-type strains are indicated in bold black type.

In this study, two *Neofusicoccum* strains were isolated from *Platycladus orientalis* in the Ming Tombs area of Beijing. Based on a multi-gene phylogenetic analysis using the concatenated sequences of the ITS, *tef1-α*, *tub2*, and *rpb2* genes, with *Botryosphaeria dothidea* (CBS 115476) selected as the outgroup (Xu et al. 2022; Si et al. 2023), a multilocus phylogenetic tree of *Neofusicoccum* isolates was reconstructed (Fig. 5), with the complete phylogeny of the genus provided in Suppl. material 3. The phylogenetic tree revealed that the two isolated strains, CFCC 72629 and CFCC 72636, clustered together with *Neofusicoccum occulatum* (CBS 128008) in the same clade, with bootstrap probability support values of 90/0.99 (ML/BI) (Fig. 5). The phylogenetic tree contained a total of 1887 characters, comprising 1610 constant characters, 203 variable characters, and 74 parsimony-informative characters. In the ML analysis based on the combined gene dataset, the matrix possessed 274 distinct alignment patterns. Estimated base frequencies are as follows: A = 0.217461, C = 0.289355, G = 0.277682, T = 0.215502, AC = 1.500488, AG = 4.266001, AT = 1.011175, CG = 1.409411, CT = 8.476073, GT = 1.000000, and gamma distribution shape parameter: α = 0.170140. The phylogenetic trees constructed using ML and BI exhibited identical topology.

#### ﻿Phylogenetic analyses of Chaetomiaceae

**Figure 6.**
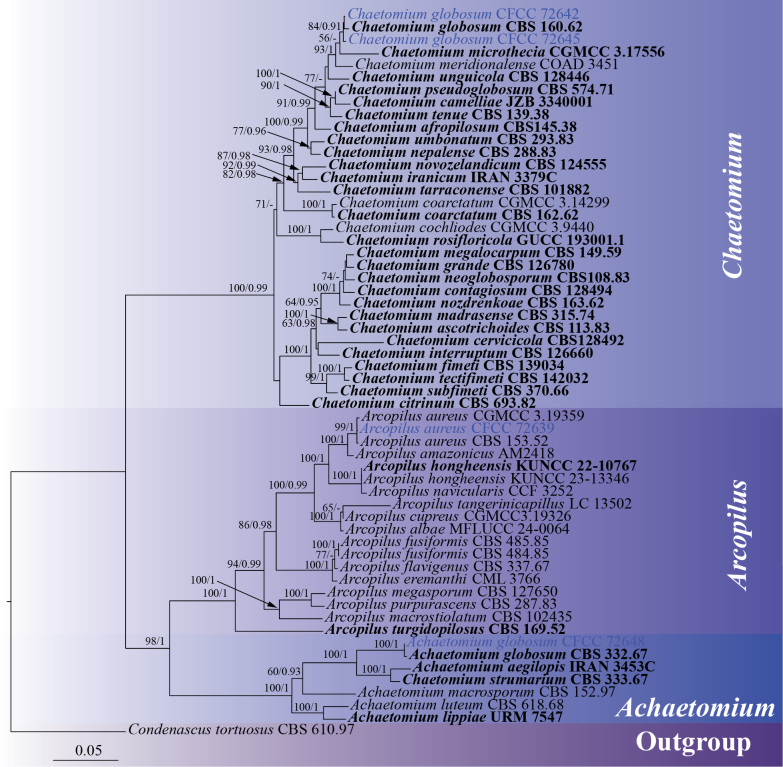
ML phylogenetic tree of *Achaetomium*, *Arcopilus*, and *Chaetomium* based on combined ITS, LSU, *rpb2*, and *tub2* sequence data. The tree is rooted with *Condenascus tortuosus* (CBS 610.97). Bootstrap support values from ML analysis (ML ≥ 50%) and Bayesian posterior probabilities (BI ≥ 0.90) are shown at the nodes. Strains obtained in this study are marked in blue; ex-type strains are indicated in bold black type.

In this study, four Chaetomiaceae strains representing three genera and three known species (*Achaetomium globosum*, *Arcopilus aureus*, and *Chaetomium globosum*) were isolated from *Platycladus orientalis*. A multigene phylogenetic tree of *Achaetomium*, *Arcopilus*, and *Chaetomium* was constructed using concatenated sequences of the ITS, LSU, *rpb2*, and *tub2* genes. Reference sequences were sourced from previously published studies (Condé et al. 2023; Zhang et al. 2024b; Qian et al. 2024), with *Condenascus tortuosus* (CBS 610.97) designated as the outgroup (Qian et al. 2024). The phylogenetic tree revealed that the four strains formed three distinct clades: strain CFCC 72648 clustered with *Achaetomium globosum* (CBS 332.67) with strong support values of 100/1 (ML/BI); strain CFCC 72639 clustered with *Arcopilus aureus* with strong support values of 99/1 (ML/BI); and strains CFCC 72642 and CFCC 72645 clustered with *Chaetomium globosum* (CBS 160.62) with support values of 84/0.91 (ML/BI) (Fig. 6). The phylogenetic tree contained a total of 2551 characters, comprising 1648 constant sites, 166 variable sites, and 737 parsimony-informative characters. In the ML analysis based on the combined gene dataset, the matrix possessed 1098 distinct alignment patterns. Estimated base frequencies are as follows: A = 0.230360, C = 0.282247, G = 0.281712, T = 0.205681, AC = 1.245731, AG = 3.563794, AT = 1.038175, CG = 1.366404, CT = 5.728326, GT = 1.000000, and gamma distribution shape parameter: α = 0.216082. The phylogenetic trees constructed using ML and BI exhibited identical topology.

#### ﻿Phylogenetic analyses of *Seiridium*

**Figure 7.**
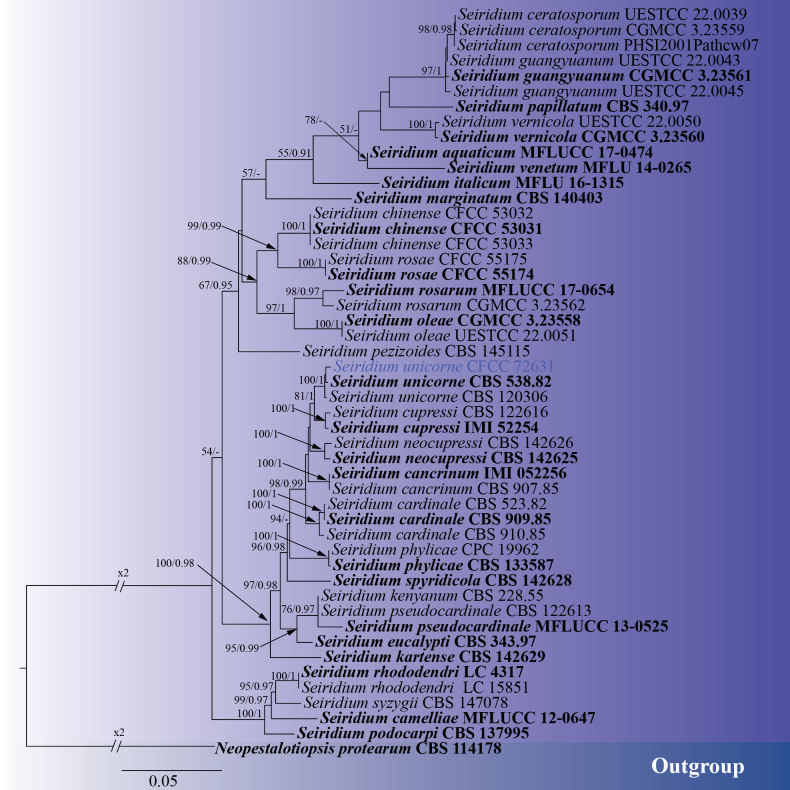
ML phylogenetic tree of *Seiridium* based on combined ITS, LSU, *rpb2*, *tef1-α*, and *tub2* sequence data. The tree is rooted with *Neopestalotiopsis protearum* (CBS 114178). Bootstrap support values from ML analysis (ML ≥ 50%) and Bayesian posterior probabilities (BI ≥ 0.90) are shown at the nodes. Strains obtained in this study are marked in blue; ex-type strains are indicated in bold black type.

In this study, one *Seiridium* strain was isolated from dead twigs of *Platycladus orientalis*. A multi-gene phylogenetic tree of *Seiridium* was constructed using concatenated sequences of ITS, LSU, *rpb2*, *tef1-α*, and *tub2* genes. The relevant reference sequences were referenced from the previously published article (Li et al. 2022), with *Neopestalotiopsis protearum* (CBS 114178) designated as the outgroup (Li et al. 2022). Phylogenetic analysis revealed that strain CFCC 72631 clustered with *Seiridium unicorne* (ex-type strain CBS 538.82) with strong support values of 100/1.00 (ML/BI) (Fig. 7). The phylogenetic tree contained a total of 3660 characters, comprising 2553 constant sites, 317 variable sites, and 790 parsimony-informative characters. In the ML analysis based on the combined gene dataset, the matrix possessed 1318 distinct alignment patterns. Estimated base frequencies are as follows: A = 0.243105, C = 0.264128, G = 0.238386, T = 0.254381, AC = 0.913277, AG = 3.236090, AT = 0.909592, CG = 0.835950, CT = 4.722234, GT = 1.000000, and gamma distribution shape parameter: α = 0.226331. The phylogenetic trees constructed using ML and BI exhibited identical topology.

### ﻿Taxonomy

#### ﻿Dothideomycetes O.E. Erikss. & Winka

**Pleosporales Luttr. ex M.E. Barr**

**Didymosphaeriaceae Munk**

##### *Spegazzinia* Sacc

###### ﻿ Spegazzinia juniperi

Taxon classificationFungiPleosporalesDidymosphaeriaceae

Z.X. Bi & C.M. Tian sp. nov.

947525D1-C05A-5E79-9F1A-1D02ED1CB410

859530

Fig. 8

####### Etymology.

Named after the host genus, *Juniperus*.

####### Specimens examined.

China • Beijing City, Changping District, Dingling, Ming Tombs Scenic Area, 40°17'28"N, 116°14'31"E, on the healthy cones of *Juniperus chinensis*, 31 March 2025, Z.X. Bi, holotype BJFC-S2582, ex-type cultures CFCC 72647.

####### Description.

Isolated from healthy cones of *Juniperus chinensis*. **Sexual morph**: Not observed. **Asexual morph**: Hyphomycetous. On PDA medium, sporulation began after approximately 3 weeks of cultivation. ***Hyphae*** were initially colorless and transparent, turning brown at maturity, branched, septate, thick-walled, and smooth, 1.2–8.2 µm in diam. ***Sporodochia*** were dark brown to black, granular, dense, slightly moist, and 150–430 µm in diam. ***Conidiophore mother cells*** were subcylindrical, thin-walled, smooth, initially colorless and transparent, later pale brown, 4.3–9.9 × 2.5–5.2 (x̄ = 6.6 × 4.1 µm; n = 25) µm. ***Conidiophores*** have two types of morphology, ***Conidiophores of α conidia*** are upright or curved, light brown or dark brown, unbranched, 13.0–78.6 × 1.4–3.5 µm (x̄ = 42.3 × 2.3 µm; n = 30). ***Conidiophores of β conidia*** are colorless and transparent at the initial stage and turn light brown to dark brown after maturity, 18.4–75.5 × 1.3–3.2 µm (x̄ = 43.7 × 2.4 µm; n = 30). The ***conidia*** have two forms:***α conidia*** 18.2–28.3 × 15.8–24.3 µm (x̄ = 22.6 × 19.9 µm; n = 50), stellate, 4-celled, brown to dark brown, each cell globose to subglobose, some cells exhibit verrucose (wart-like) ornamentation and spinose projections (spines) in brown to dark brown, with spine lengths 1.8–7.6 µm, septa distinctly constricted. ***β Conidia*** 14.3–18.6 × 13.4–17.8 µm (x̄ = 16.5 × 15.6 µm; n = 50), trifoliate (clover-shaped), discoid, 4-celled, each cell slightly subtriangular, lacking spinose projections but with a finely roughened surface, initially hyaline and transparent, maturing to pale brown or dark brown, septa arranged in a near-cruciate (cross-like) pattern, with lighter pigmentation adjacent to septa and distinct constrictions at septal junctions.

**Figure 8.**
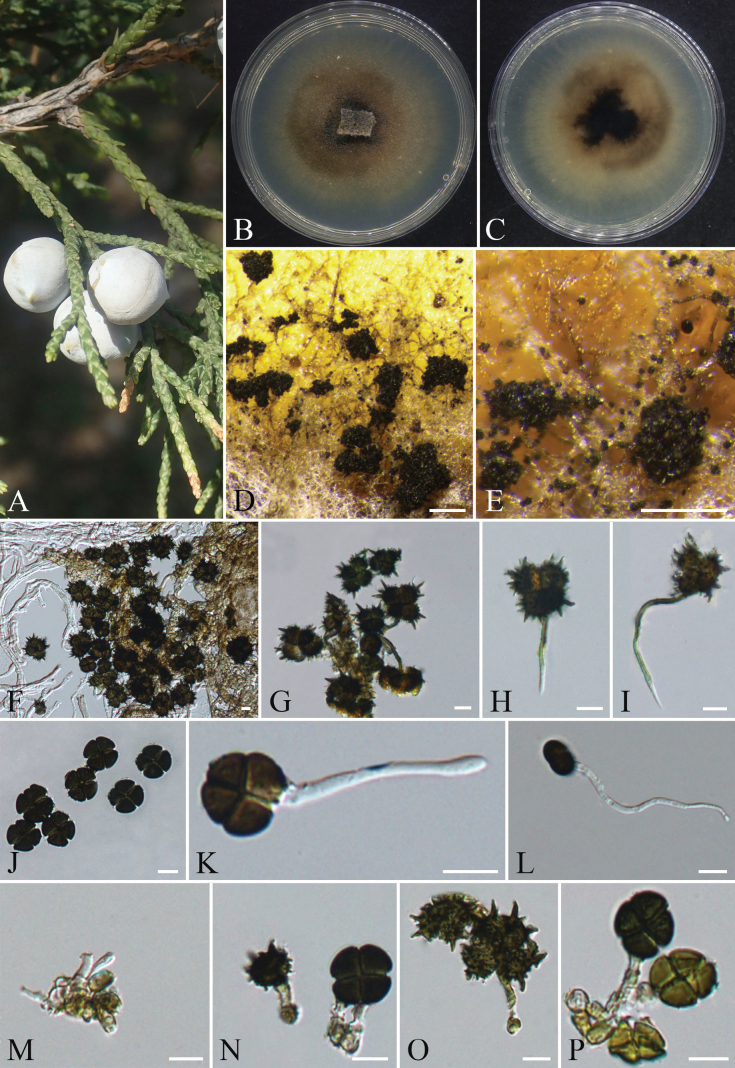
*Spegazzinia juniperi* (CFCC 72647). A. Healthy cones of the host plant *Juniperus chinensis*; B, C. Colony surface and reverse on PDA; D, E. Sporodochia on PDA; F–I. *α* conidia and *α* conidiophores; J–L. *β* conidia and *β* conidiophores; M–P. Conidiophore mother cells. Scale bars: 200 µm (D, E); 10 µm (F–P).

####### Culture characteristics.

Cultured on PDA at 25 °C under dark conditions for approximately 10 days, the colony diameter reaches about 60 mm. The initial colony appears grayish-white, exhibits radial growth, and adheres to the medium with a felt-like texture, displaying denser hyphae near the central region. By day 14, the colony develops concentric rings, the center becomes dark brownish-black, while the margin fades to light grayish-brown, with a regular edge. On the reverse side, the central area is black, transitioning outward to light brownish-black, and finally to light grayish-brown at the outermost edge. After 20 days, dark brown irregularly shaped sporodochia form in both the central and marginal areas of the colony.

####### Notes.

Phylogenetic analysis based on ITS, LSU, SSU, and *tef1-α* indicates that strains CFCC 72647 (ex-type strain) and CFCC 72641 separated from other known strains and formed a distinct clade with strong support values of 99/1 (ML/BI) (Fig. 3). This clade is clearly separated as a sister group to *Spegazzinia tessarthra* (support values ML/BI = 95/0.99) and shows close phylogenetic affinity to *S. radermacherae* (Fig. 3). Morphologically, *S. juniperi* differs from *S. tessarthra* and *S. radermacherae* in having granular, slightly moist sporodochia. The *α*-conidia of *S. juniperi* are larger than those of *S. tessarthra* (18.2–28.3 × 15.8–24.3 µm vs. 15–20 × 14–18 µm), and its *β*-conidia are broader (13.4–17.8 µm vs. 8–12 µm) (Tennakoon et al. 2022). Compared to *S. radermacherae*, *S. juniperi* exhibits larger *α*-conidia (18.2–28.3 × 15.8–24.3 µm vs. 18–22 × 17.5–20 µm), broader *β*-conidia (13.4–17.8 µm vs. 8–10 µm), and longer spines (1.8–7.6 µm vs. 2–3 µm) (Jayasiri et al. 2019). Furthermore, this species could be differentiated from *S. tessarthra* (SH 287) at the ITS, LSU, SSU, and *tef1-α* loci with nucleotide differences of 2/355 bp in ITS, 7/890 bp in LSU, 12/925 bp in *tef1*, and 1/1008 bp in SSU. It was distinguishable from *S. radermacherae* (MFLUCC 17-2285) at the ITS and *tef1-α* loci, showing 4/355 bp differences in ITS and 73/925 bp in *tef1-α*. Therefore, based on phylogenetic and morphological data, *S. juniperi* collected from *Juniperus chinensis* is formally described as a new species.

#### ﻿Sordariomycetes O.E. Erikss. & Winka

**Xylariales Nannf**

**Apiosporaceae K.D. Hyde, J. Fröhl., Joanne E. Taylor & M.E. Barr**

##### *Nigrospora* Zimm

###### ﻿ Nigrospora platycladiensis

Taxon classificationFungiXylarialesApiosporaceae

Z.X. Bi & C.M. Tian sp. nov.

444A481F-254D-5025-91A7-7D0AEC6B3FF2

859531

Fig. 9

####### Etymology.

Named after the host genus, *Platycladus*.

####### Specimens examined.

China • Beijing City, Changping District, Ming Tombs Reservoir, 40°14'57"N, 116°15'54"E, on the discolored scale leaves of *Platycladus orientalis*, 23 February 2025, Z.X. Bi, holotype BJFC-S2578, ex-type strain CFCC 72632.

####### Description.

**Sexual morph**: Not observed. **Asexual morph: *Hyphae*** Intertwined, hyaline to pale brownish, slightly thick-walled, smooth-surfaced, septate, branched, 1.6–5.1 µm in diam. ***Conidiophores*** reduced to conidiogenous cells. ***Conidiogenous cells*** Initially hyaline, becoming pale brown with age, solitary or aggregated in clusters, ampulliform to subcylindrical, 5.5–8.9 × 3.9–6.9 µm (av. ± S.D. = 7.3 ± 0.9 × 5.3 ± 0.8; n = 30). ***Conidia*** mostly solitary and sparse, but capable of forming clusters under pine needle induction, initially hyaline, turning black to brown at maturity, smooth-walled, aseptate, subglobose, 10.4–17.5 × 9.7–17.3 µm (av. ± S.D.= 14.68 ± 1.75 × 13.6 ± 2.0; n = 50).

####### Culture characteristics.

When cultured on PDA at 25 °C under dark conditions for 7 days, the colony diameter reaches 60 mm. The colony appears fluffy with well-developed aerial hyphae. These hyphae later intertwine to form small aggregates. Initially white, the colony begins to produce light yellow hyphae after 10 days. The reverse side of the colony is pale brownish. After 20 days, brownish block-like spots start to develop on the reverse. By 30 days, deep black, irregular patches form near the bottom of the medium.

**Figure 9.**
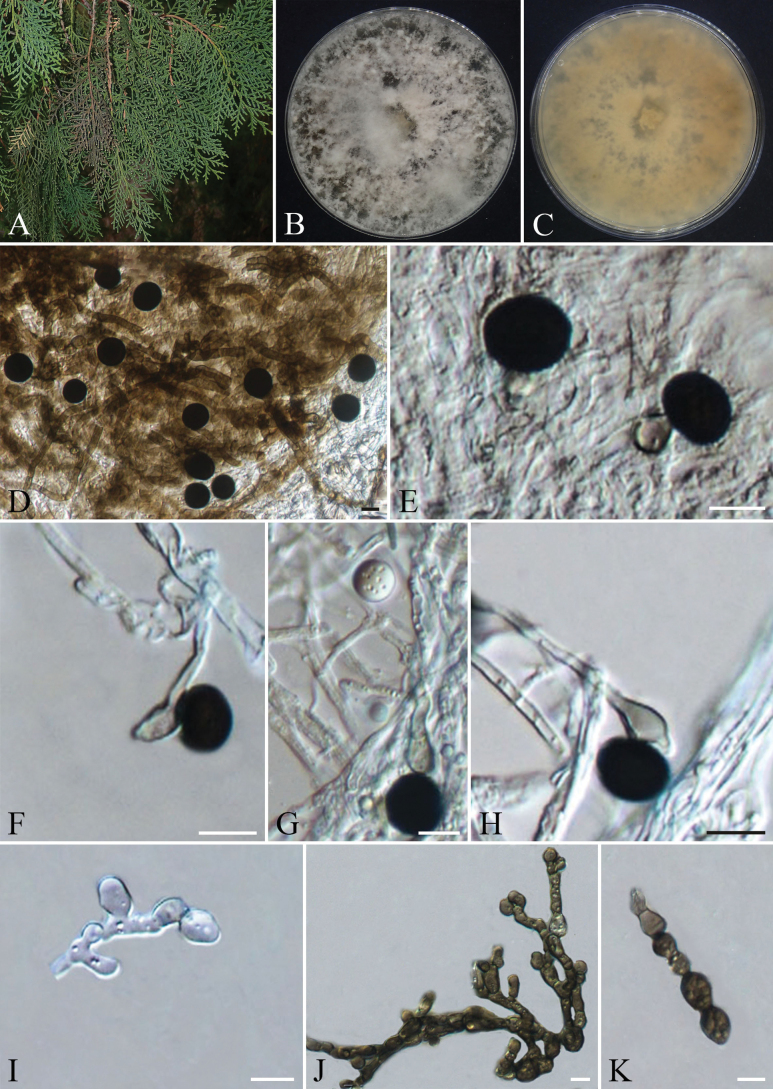
*Nigrospora platycladiensis* (CFCC 72632). A. Diseased scale leaves habit of *Platycladus orientalis*; B, C. Colony surface and reverse on PDA; D. Conidia; E–I. Conidiogenous cells; J, K. Hyphae growing at the bottom of PDA. Scale bars: 10 µm (D–K).

####### Notes.

Phylogenetic analysis based on ITS, *tub2*, and *tef1-α* loci revealed that strains CFCC 72630 and CFCC 72632 (ex-type strain) formed a distinct clade with strong statistical support of 100/1 (ML/BI) and clustered as a sister clade to *Nigrospora guangdongensis* (ex-type strain CFCC 53917) with bootstrap support values of 98/1 (ML/BI) (Fig. 2). However, this species can be distinguished from *N. guangdongensis* by nucleotide differences at the ITS, *tef1*, and *tub2* loci (1/534 bp with 4 gaps in ITS, 7/408 bp with 7 gaps in *tub2*, 36/495 bp with 10 gaps in *tef1*). Morphologically, the newly discovered species *N. platycladiensis* from *Platycladus orientalis* showed partial overlap in conidial size with its closely related species *N. guangdongensis* (10.4–17.5 μm vs. 13.6–20.9 μm). However, the average conidial length of *N. platycladiensis* was significantly smaller than that of *N. guangdongensis* (av. ± S.D. = 14.6 ± 1.7 μm vs. av. ± S.D. = 16.8 ± 1.9 μm). Additionally, the conidiogenous cells of *N. platycladiensis* were markedly narrower than those of *N. guangdongensis* (3.9–6.9 μm vs. 7.1–9.9 μm) (Tian et al. 2020). Based on integrated phylogenetic and morphological data, *Nigrospora platycladiensis* is proposed as a novel species.

#### ﻿Dothideomycetes O.E. Erikss. & Winka

**Botryosphaeriales C.L. Schoch, Crous & Shoemaker**

**Aplosporellaceae Slippers, Boissin & Crous**

##### *Aplosporella* Speg

*Aplosporella* was introduced by Spegazzini (1880), with *Aplosporella chlorostroma* designated as its type species. The type species defines the genus as characterized by multilocular conidiomata that produce brown, aseptate, verrucose conidia with filiform paraphyses (Damm et al. 2007). In this study, five fungal strains were isolated from dead twigs and healthy strobili of *Platycladus orientalis*, belonging to three species: *A. hesperidica*, *A. javeedii*, and *A. prunicola*.

###### ﻿ Aplosporella hesperidica

Taxon classificationFungiBotryosphaerialesAplosporellaceae

Speg., Anal. Soc. cient. argent. 13(1): 18 (1882).

0C82162F-BEEE-5372-AAC6-A9ACCA01DDE4

Fig. 10

####### Description.

**Sexual form**: Not observed. **Asexual form: *Fruiting bodies*** distributed on dead twigs of *Platycladus orientalis*, mostly breaking through the host epidermis, appearing brown-black or gray-black. ***Conidiomata*** pycnidial, immersed or semi-immersed, light brown, solitary, multiloculate, 205–588 µm diam., the outer wall composed of light brown textura angularis, gradually becoming lighter inward, with the inner region hyaline. ***Ostiole*** central, black or dark brown, 41.7–57.1 µm diam. ***Conidiophores*** reduced to conidiogenous cells. ***Conidiogenous cells*** smooth, hyaline, nearly cylindrical, thin-walled, 5.8–11.9 × 1.8–3.5 µm (av. ± S.D. = 8.4 ± 2.1 × 2.4 ± 0.5). ***Paraphyses*** long-cylindrical, 31.4–87.1 × 1.6–4.9 µm, hyaline, thin-walled, smooth, occasionally branched at the base. ***Conidia*** initially hyaline with a truncate base, turning brown or black at maturity, aseptate, subellipsoid or broadly ellipsoid, 14.1–22.2 × 8.1–15.6 µm (av. ± S.D. = 16.7 ± 1.8 × 10.8 ± 1.4).

####### Culture characteristics.

On PDA at 25 °C under dark conditions for approximately 7 days, colonies reach a diameter of 60 mm. Initially white, the colonies exhibit radial growth patterns. The aerial mycelium appears appressed to floccose, ranging in color from white to smoke-grey. Mycelial density shows regional variation—being relatively sparse near the central region while becoming more densely distributed towards the marginal zone.

**Figure 10.**
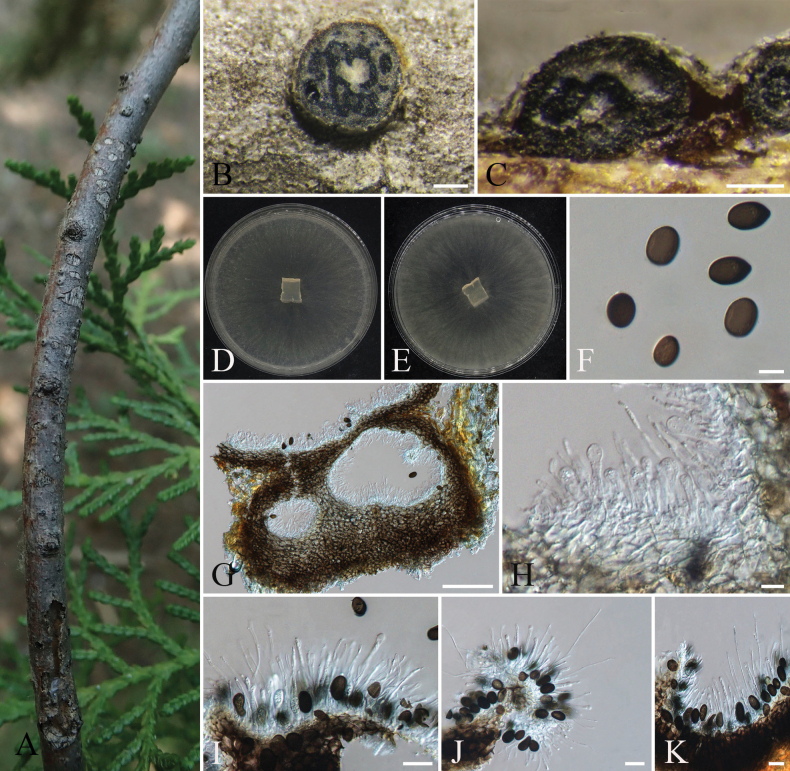
*Aplosporella hesperidica* (CFCC 72635). A. Conidiomata on a dead twig of *Platycladus orientalis*; B. Cransverse section of conidioma; C. Longitudinal section of conidioma; D, E. Colony morphology on PDA front and reverse views; F. Conidia; G. Pycnidia; H–K. Conidiogenous cells and paraphyses. Scale bars: 200 µm (B, C); 100 µm (G); 20 µm (I–K); 10 µm (H).

####### Specimens examined.

China • Beijing City, Changping District, Ming Tombs Reservoir, 40°14'52"N, 116°15'30"E, on the dead branches of *Platycladus orientalis*, 2 October 2024, Z.X. Bi, BJFC-S2566, living culture CFCC 72635.

####### Notes.

*Aplosporella hesperidica* was first discovered on Citrus × aurantium in Argentina (Spegazzini 1882). Subsequently, Dissanayake et al. (2021) reported its first occurrence in China, followed by Lin et al. (2023b) detecting this fungal species on *Euonymus japonicus*. Additionally, *A. hesperidica* has been found to cause stem rot in cowpea in India (Deepika et al. 2020). Comprehensive phylogenetic and morphological analyses identified the fungal strain CFCC 72635 as *A. hesperidica*. This is the first report of *A. hesperidica* on *Platycladus orientalis*.

###### ﻿ Aplosporella javeedii

Taxon classificationFungiBotryosphaerialesAplosporellaceae

Jami, Gryzenh., Slippers & M.J. Wingf., Fungal Biology 118(2): 174 (2013)

08D44C84-9AEA-5F3D-9921-89E088215CEB

Fig. 11

####### Description.

**Sexual form**: Not observed. **Asexual form**: Sporulation began after 2 weeks of cultivation on PDA medium. ***Conidiomata*** pycnidial, immersed to semi-immersed, grey-olivaceous, solitary, subglobose, 529–883 µm diam., pycnidial wall consists of dark brown textura angularis in the outer layers, gradually becoming paler in coloration towards the interior, with the innermost layers thinning and becoming hyaline and transparent. ***Conidiophores*** reduced to conidiogenous cells. ***Conidiogenous cells*** smooth, hyaline, elongate-ellipsoidal, thin-walled, gradually tapering toward the apex, 9.3–18.0 × 2.0–6.8 μm (av. ± S.D. = 12.2 ± 3.0 × 3.6 ± 1.1). ***Paraphyses*** long-cylindrical, 22.9–37.2 × 2.0–5.0 µm, hyaline, thin-walled, smooth, occasionally branched. ***Conidia*** initially hyaline, gradually turning pale brown to yellowish-brown, and finally dark brown at maturity, aseptate, ellipsoidal, 18.3–22.2 × 6.8–9.0 µm (av. ± S.D. = 19.9 ± 1.2 × 7.9 ± 0.5).

####### Cultural characteristics.

On PDA at 25 °C under dark conditions, the colony reached approximately 60 mm in diameter after 7 days of incubation. The aerial mycelium was well-developed, appearing floccose and whitish-gray with sparse growth in the central region and denser growth at the periphery. After 20 days, the colony developed an olivaceous coloration, with abundant grayish-green aerial mycelium particularly concentrated near the marginal zone.

**Figure 11.**
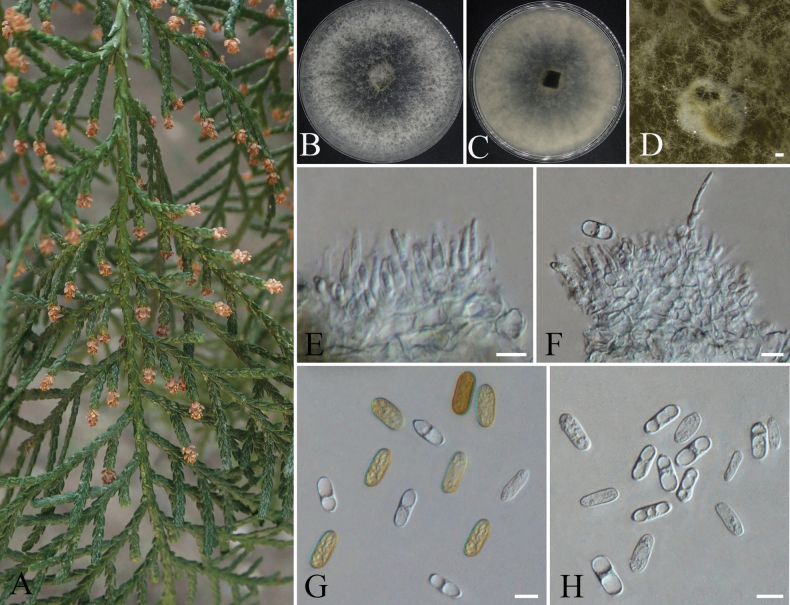
*Aplosporella javeedii* (CFCC 72643). A. Healthy strobili of *Platycladus orientalis*; B, C. Colony morphology on PDA front and reverse views; D. The conidiomata on PDA; E, F. Conidiogenous cells and paraphyses; G, H. Conidiogenous cells and conidia. Scale bars: 200 µm (D); 10 µm (E–H).

####### Specimens examined.

China • Beijing City, Changping District, Dingling, Ming Tombs Scenic Area, 40°17'23"N, 116°14'8"E, on the dead branches of *Platycladus orientalis*, 21 September 2024, Z.X. Bi, BJFC-S2567, living culture CFCC 72633; China • Beijing City, Changping District, Dingling, Ming Tombs Scenic Area, 40°17'28"N, 116°14'31"E, on the healthy strobili of *P. orientalis*, 31 March 2025, Z.X. Bi, BJFC-S2568, living culture CFCC 72643.

####### Notes.

*Aplosporella javeedii* was first described by Jami et al. (2013) and isolated from healthy branches of *Celtis africana* and *Searsia lancea*. Fan et al. (2015) subsequently reported its first occurrence in China, where it was isolated from five host plants, including *Juniperus chinensis*, exhibiting stem canker symptoms. Additionally, *A. javeedii* has been identified as the causal agent of mulberry (*Morus alba*) branch blight disease (Jia et al. 2019). According to literature records, this fungal species has now been documented across more than 10 plant families (Fan et al. 2015; Zhu et al. 2018; Pan et al. 2019; Lin et al. 2023b; Wu et al. 2024). Based on comprehensive phylogenetic and morphological analyses, strains CFCC 72633 and CFCC 72643 were identified as *A. javeedii*.

###### ﻿ Aplosporella prunicola

Taxon classificationFungiBotryosphaerialesAplosporellaceae

Damm & Crous, Fungal Diversity 27: 39 (2007).

5C06E227-F085-57E5-87E9-EDF71B77F768

Fig. 12

####### Description.

**Sexual form**: Not observed. **Asexual form: *Fruiting bodies*** densely distributed on dead twigs of *Platycladus orientalis*, mostly immersed in the host epidermis. ***Conidiomata*** pycnidial, immersed, multilocular, solitary, 406–651 μm diam., pycnidial wall composed of dark brown textura angularis in the outer layers, gradually becoming paler toward the interior, with the innermost region thin-walled and hyaline. ***Ostiole*** central, 67–122 μm diam. ***Conidiophores*** reduced to conidiogenous cells. ***Conidiogenous cells*** cylindrical, smooth, hyaline, 6.7–14.6 × 2.1–5.9 μm (av. ± S.D. = 10.5 ± 2.5 × 3.7 ± 1.1). ***Paraphyses*** long-cylindrical, occasionally swollen at the apex, 30.7–92.5 × 1.1–7.0 μm, septate, hyaline, smooth-walled, and branched. ***Conidia*** 16.1–23.0 × 8.3–14.2 μm (av. ± S.D. = 19.8 ± 1.6 × 11.1 ± 1.3), initially hyaline, gradually turning yellowish-brown, and finally dark brown at maturity, aseptate, smooth-walled.

####### Cultural characteristics.

On PDA at 25 °C under dark conditions, the colony reached approximately 60 mm in diameter after 4 days of incubation, exhibiting abundant floccose aerial mycelium. After 10 days, the colony developed pale grayish-green pigmentation, later transitioning to whitish-gray and ultimately olivaceous. Sporulation commenced after 2 weeks, forming subglobose pycnidia.

**Figure 12.**
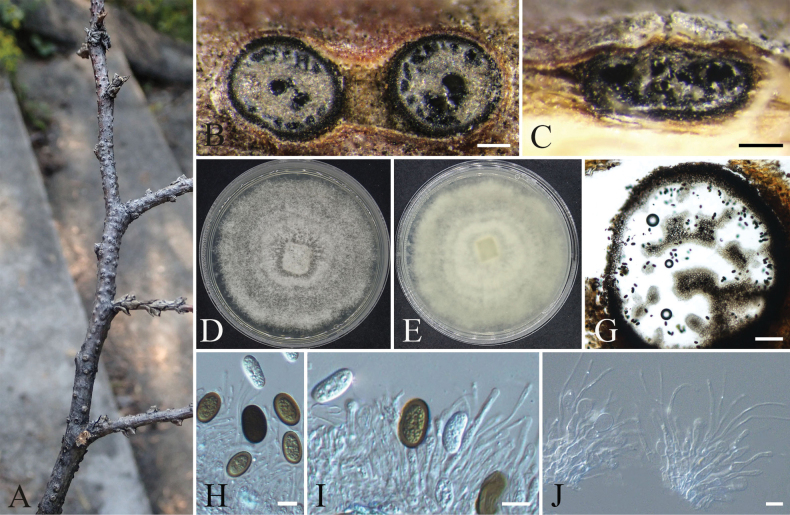
*Aplosporella prunicola* (CFCC 72634). A. Conidiomata on a dead twig of *Platycladus orientalis*; B. Transverse section of conidioma; C. Longitudinal section of conidioma; D, E. Colony morphology on PDA front and reverse views; G. Pycnidia; H. Conidia; I, J. Conidiogenous cells and paraphyses. Scale bars: 200 µm (B, C); 100 µm (G); 10 µm (H–J).

####### Specimens examined.

China • Beijing City, Changping District, Longshan Sub-Farm, Ming Tombs, 40°14'25"N, 116°13'17"E, on the dead branches of *Platycladus orientalis*, 18 July 2024, Z.X. Bi & C.M. Tian, BJFC-S2569, living culture CFCC 72634; China • Beijing City, Changping District, Dayu Mountain Scenic Area, Ming Tombs, 40°18'32"N, 116°11'47"E, on the dead branches of *P. orientalis*, 23 October 2024, Z.X. Bi & M.H. Wang, BJFC-S2570, living culture CFCC 72640.

####### Notes.

*Aplosporella prunicola* was first isolated by Damm et al. (2007) from Prunus persica var. nucipersica in South Africa. In China, *A. prunicola* has been recorded on *Castanea mollissima*, *Euonymus japonicus*, and *Zanthoxylum bungeanum* (Jiang 2021; Li et al. 2023; Lin et al. 2023b). Based on comprehensive phylogenetic and morphological analyses, strains CFCC 72634 and CFCC 72640 were identified as *A. prunicola*.

#### ﻿Dothideomycetes O.E. Erikss. & Winka

**Botryosphaeriales C.L. Schoch, Crous & Shoemaker**

**Botryosphaeriaceae Theiss. & Syd**

##### *Neofusicoccum* Crous, Slippers & A.J.L. Phillips

*Neofusicoccum* was established by Crous et al. (2006), with *Neofusicoccum parvum* designated as its type species. Members of this genus commonly exist as endophytes, saprobes, or latent pathogens across diverse host plants, being particularly notorious for causing dieback and canker diseases in woody hosts (Crous et al. 2006; Brewer et al. 2021; Guo 2023). These fungi predominantly reproduce asexually, with their sexual morph being relatively uncommon (Pennycook and Samuels 1985; Lopes et al. 2017). The genus exhibits a cosmopolitan distribution with a broad host range (Crous et al. 2006; Brewer et al. 2021). In the present study, two fungal strains isolated from withered branches of *Platycladus orientalis* were identified as *N. occulatum*. According to previous reports, this species of fungi is the pathogen causing canker and branch blight of cypress (Liu et al. 2022; Guo 2023).

###### ﻿ Neofusicoccum occulatum

Taxon classificationFungiBotryosphaerialesBotryosphaeriaceae

Sakalidis & T.Burgess, Molecular Phylogenetics and Evolution 60(3): 340(2011)

2D8E0FE4-17E5-519F-893B-516B362AC02B

Fig. 13

####### Description.

**Sexual morph**: Not observed. **Asexual morph: *Fruiting bodies*** densely distributed on dead twigs of *Platycladus orientalis*. ***Conidiomata*** pycnidial immersed in bark surface, aggregated, unilocular or multilocular, subglobose, black, 58–194 µm diam. ***Conidiophores*** reduced to conidiogenous cells. ***Conidiogenous cells*** thin-walled, hyaline, ovoid to cylindrical, 6.1–19.5 × 1.0–4.3 µm (av. ± S.D. = 13.3 ± 3.7 × 2.8 ± 0.9). ***Conidia*** unicellular, hyaline, fusiform to subellipsoid, containing granular inclusions, occasionally with 1–2 oil droplets, 14.0–22.9 × 4.3–8.1 µm (av. ± S.D. = 19.8 ± 1.9 × 6.1 ± 0.7).

####### Cultural characteristics.

On PDA at 25 °C under dark conditions, colonies reached approximately 60 mm in diameter after 7 days of incubation, exhibiting dense, floccose mycelium. After 10 days, the aerial hyphae developed a smoke-gray coloration, while the reverse side of colonies turned grayish-brown. With prolonged cultivation, the mycelium darkened to blackish-brown, accompanied by black pigmentation on the colony reverse. At approximately 20 days, grayish-white to smoke-black pycnidia formed on the medium, often embedded in mycelial mats and appearing as irregular masses or subglobose structures. At maturity, these pycnidia produced pale yellow conidial masses.

**Figure 13.**
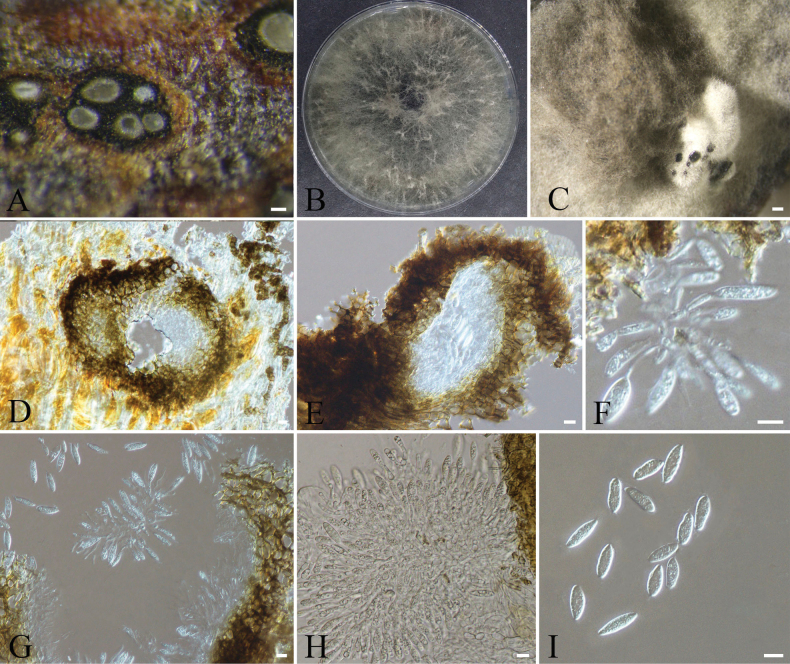
*Neofusicoccum occulatum* (CFCC 72629). A. Conidiomata on withered twigs of *Platycladus orientalis*; B. Colony morphology on PDA front views; C. The conidiomata on PDA; D, E. Pycnidia; F–H. Conidiogenous cells; I. Conidia. Scale bars: 100 µm (C); 10 µm (D–I).

####### Specimens examined.

China • Beijing City, Changping District, Mangshan National Forest Park, Ming Tombs, 40°16'5"N, 116°16'51"E, on the diseased branches of *Platycladus orientalis*, 23 November 2024, Z.X. Bi & W.K. Gao, BJFC-S2579, living culture CFCC 72629; China • Beijing City, Changping District, Mangshan National Forest Park, Ming Tombs, 40°16'5"N, 116°16'57"E, on the dead branches of *P. orientalis*, 23 November 2024, Z.X. Bi & W.K. Gao, BJFC-S2580, living culture CFCC 72636.

####### Notes.

*Neofusicoccum occulatum* was introduced by Sakalidis et al. (2011) and was isolated from *Eucalyptus* spp. and *Wollemia nobilis* in Australia. Previous studies have demonstrated that this fungus is a pathogen causing canker and shoot blight in *Platycladus orientalis* (Liu et al. 2022; Guo 2023). It has also been recorded on host plants such as *Prunus persica* and *Dendrobium chrysanthum* (Ma et al. 2021; Zhou et al. 2024). Based on comprehensive phylogenetic and morphological analyses, strains CFCC 72629 and CFCC 72636 were identified as *Neofusicoccum occulatum*.

#### ﻿Sordariomycetes O.E. Erikss. & Winka

**Xylariales Nannf**

**Apiosporaceae K.D. Hyde, J. Fröhl., Joanne E. Taylor & M.E. Barr**

##### *Nigrospora* Zimm

###### ﻿ Nigrospora oryzae

Taxon classificationFungiXylarialesApiosporaceae

(Berk. & Broome) Petch, J. Indian Bot. Soc. 4: 24 (1924)

A73F79DD-C46D-5C0E-8416-38001906FF50

Fig. 14

####### Description.

**Sexual morph**: Not observed. **Asexual morph: *Hyphae*** interwoven, initially hyaline, becoming brownish with age, septate, frequently branched, 2.4–6.7 µm diam. ***Conidiophores*** reduced to conidiogenous cells. ***Conidiogenous cells*** predominantly clustered but occasionally solitary on hyphae, hyaline, ampulliform to subglobose, 3.1–13.8 × 3.4–7.4 µm (av. ± S.D. = 7.3 ± 2.2 × 5.5 ± 1.0). ***Conidia*** typically aggregated in slimy masses, initially white and hyaline, gradually turning pale brown, and finally black at maturity, smooth-walled, aseptate, globose to subellipsoid, 11.7–14.8 × 10.2–13.9 µm (av. ± S.D. = 12.8 ± 0.6 × 11.9 ± 0.9).

####### Cultural characteristics.

On PDA medium, colonies initially appeared white and cottony. After 7 days of incubation, the mycelium developed a smoke-gray coloration, with denser growth and darker pigmentation in the central region compared to the margins. By day 20, the colonies turned grayish-black throughout.

**Figure 14.**
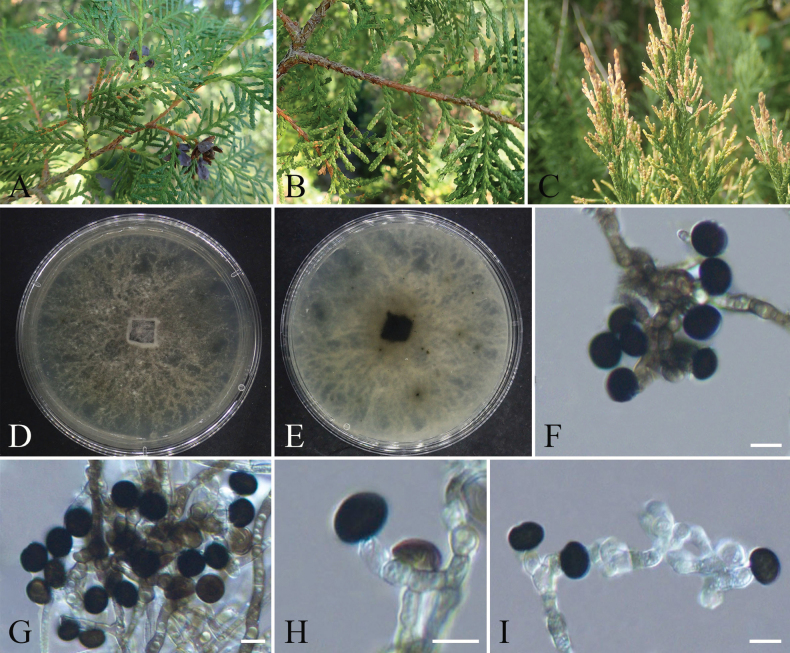
*Nigrospora oryzae* (CFCC 72638, CFCC 72644). A, B. Diseased scale leaves of *Platycladus orientalis*; C. Withered leaf tips of *Juniperus procumbens*; D, E. Colony surface and reverse on PDA; F, G. Conidia; H, I. Conidiogenous cells. Scale bars: 10 µm (F–I).

####### Specimens examined.

China • Beijing City, Changping District, Dayu Mountain Scenic Area, Ming Tombs, 40°18'20"N, 116°12'4"E, on the diseased scale leaves with lesions of *Platycladus orientalis*, 23 October 2024, Z.X. Bi & M.H. W, BJFC-S2574, living culture CFCC 72644. China • Beijing City, Changping District, Changling Scenic Area, Ming Tombs, 40°17'41"N, 116°14'24"E, on the withered leaf tips of *Juniperus procumbens*, 23 October 2024, Z.X. Bi, BJFC-S2575, living culture CFCC 72638.

####### Notes.

*Nigrospora oryzae* is recognized as both an endophyte and a pathogen causing leaf spot disease on rice (*Oryza sativa*); it commonly colonizes diverse plants and plant debris in dual roles as a pathogen and endophyte (Wang et al. 2017; Liu et al. 2021; Liu et al. 2024). In this study, two fungal strains, CFCC 72638 and CFCC 72644, were isolated from *Platycladus orientalis* and *Juniperus procumbens*. Based on comprehensive phylogenetic and morphological analyses, strains CFCC 72644 and CFCC 72638 were identified as *N. oryzae*.

###### ﻿ Nigrospora osmanthi

Taxon classificationFungiXylarialesApiosporaceae

Mei Wang & L. Cai, Persoonia 39: 135 (2017)

AD67D4D6-7DDF-56BF-86D8-77475A3B0298

Fig. 15

####### Description.

**Sexual morph**: Not observed. **Asexual morph: *Hyphae*** interwoven, initially hyaline, becoming pale brown to yellowish-brown with age, thick-walled, septate, frequently branched, 2.0–5.1 µm diam. ***Conidiophores*** reduced to conidiogenous cells. ***Conidiogenous cells*** solitary on hyphae, smooth-walled, hyaline turning pale yellowish-brown with maturation, variable in shape (phialidic, short-clavate, subglobose to cylindrical), 7.8–13.7 × 4.1–7.8 µm (av. ± S.D. = 8.2 ± 3.0 × 5.5 ± 1.1). ***Conidia*** solitary, initially hyaline, maturing to black, smooth-walled, aseptate, subglobose, 12.0–15.2 × 7.9–14.4 µm (av. ± S.D. = 13.5 ± 0.8 × 11.7 ± 1.3).

####### Cultural characteristics.

On PDA medium, colonies initially appeared white and cottony with abundant aerial mycelium, spreading radially to form concentric rings. Three distinct pigmentation zones were observed from the surface view, exhibiting a darker central region. At 10 days, the central zone developed a smoke-gray coloration while the margins gradually faded to whitish-gray. By 20 days, the entire colony turned uniformly grayish-black, maintaining a cottony, appressed growth habit across the agar surface.

**Figure 15.**
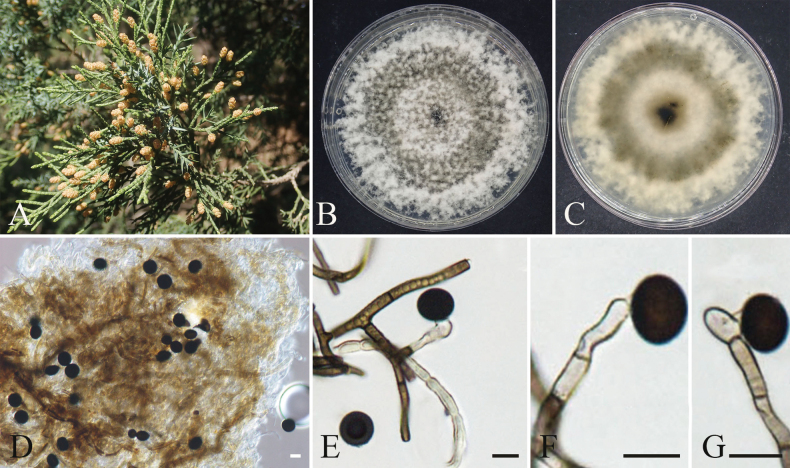
*Nigrospora osmanthi* (CFCC 72646). A. Healthy strobili of *Juniperus chinensis*; B, C. Colony surface and reverse on PDA; D. Conidia; E–G. Conidiogenous cells. Scale bars: 10 µm (D–G).

####### Specimens examined.

China • Beijing City, Changping District, Dingling Scenic Area, Ming Tombs, 40°17'28"N, 116°14'31"E, on the healthy strobili of *Juniperus chinensis*, 31 March 2025, Z.X. Bi, BJFC-S2577, living culture CFCC 72646; China • Beijing City, Changping District, Dayu Mountain Scenic Area, Ming Tombs, 40°18'20"N, 116°12'4"E, on the diseased scale leaves with lesions of *Platycladus orientalis*, 23 October 2024, Z.X. Bi & M.H. W, BJFC-S2576, living culture CFCC 72649.

####### Notes.

*Nigrospora osmanthi* was first described by Wang et al. (2017) based on specimens isolated from *Osmanthus* sp. Subsequent studies have documented its occurrence on diverse host plants, including *Cirsium setosum*, *Codium* sp., *Fagopyrum tataricum*, *Phyllostachys nigra*, *Phragmites australis*, *Rosa chinensis*, *Rudbeckia hirta*, and *Ulva* sp. (Hao et al. 2020; Shen et al. 2021; Lee et al. 2023). Based on comprehensive phylogenetic and morphological analyses, strains CFCC 72646 and CFCC 72649 were identified as *N. osmanthi*.

###### ﻿ Nigrospora philosophiae-doctoris

Taxon classificationFungiXylarialesApiosporaceae

Raza, Qian Chen & L. Cai, Studies in Mycology 101: 491(2022)

F94F37B9-6A35-55E8-9A23-26740A485917

Fig. 16

####### Description.

**Sexual morph**: Not observed. **Asexual morph: *Hyphae*** interwoven, initially hyaline, becoming pale brown to yellowish-brown with age, darkening to light brown near sporulating regions, septate, thick-walled, frequently branched, 1.5–4.8 µm in diameter. ***Conidiophores*** reduced to conidiogenous cells. ***Conidiogenous cells*** initially hyaline, maturing to pale brown or yellowish-brown, predominantly solitary but occasionally clustered (2–3 cells), phialidic or subglobose, 2.5–11.4 × 3.0–8.1 µm (av. ± S.D. = 8.1 ± 2.2 × 7.1 ± 2.0). ***Conidia*** borne singly on hyphae, rarely in sparse clusters, initially light yellowish-brown, turning black or dark brown at maturity, smooth-walled, aseptate, subglobose to ellipsoidal, 13.7–18.9 × 10.4–17.8 µm (av. ± S.D. = 16.3 ± 1.1 × 13.4 ± 1.9).

####### Specimens examined.

China • Beijing City, Changping District, Dingling Scenic Area, Ming Tombs, 40°17'28"N, 116°14'31"E, on the healthy strobili of *Juniperus chinensis*, 31 March 2025, Z.X. Bi, BJFC-S2577, living culture CFCC 72628; China • Beijing City, Changping District, Dayu Mountain Scenic Area, Ming Tombs, 40°18'20"N, 116°12'4"E, on the diseased scale leaves with lesions of *Platycladus orientalis*, 23 October 2024, Z.X. Bi & M.H. W, BJFC-S2576, living culture CFCC 72637.

####### Notes.

*Nigrospora philosophiae-doctoris* was first isolated from *Disporum sessile* (Colchicaceae) (Chen et al. 2022). Petrović et al. (2023) demonstrated that *N. philosophiae-doctoris* is a causal agent of olive leaf spot disease. Subsequently, Yan et al. (2025) isolated this species from lesions on *Camellia japonica* and reported it as a novel pathogen causing *camellia* leaf spot. Phylogenetic analyses confirmed that the two strains isolated in this study, CFCC 72628 and CFCC 72637, clustered within the same clade as *N. philosophiae-doctoris* (ex-type strain CGMCC 3.20540) with strong statistical support (ML/BI = 100/1) (Fig. 2). Morphologically, these strains exhibited conidiophores reduced to conidiogenous cells, mostly solitary, 2.5–11.4 × 3.0–8.1 µm (literature range: 4–9.5 × 3–7.5 µm); conidia were black, subglobose, and measured 13.7–18.9 × 10.4–17.8 µm (literature range: 11–16 × 8–14 µm), although slightly larger than those described by Chen et al. (2022), but all other morphological characteristics aligned with those described by Chen et al. (2022). Based on integrated phylogenetic and morphological evidence, these strains were conclusively identified as *N. philosophiae-doctoris*. This represents the first report of this fungal species on *Juniperus chinensis* and *Platycladus orientalis*.

**Figure 16.**
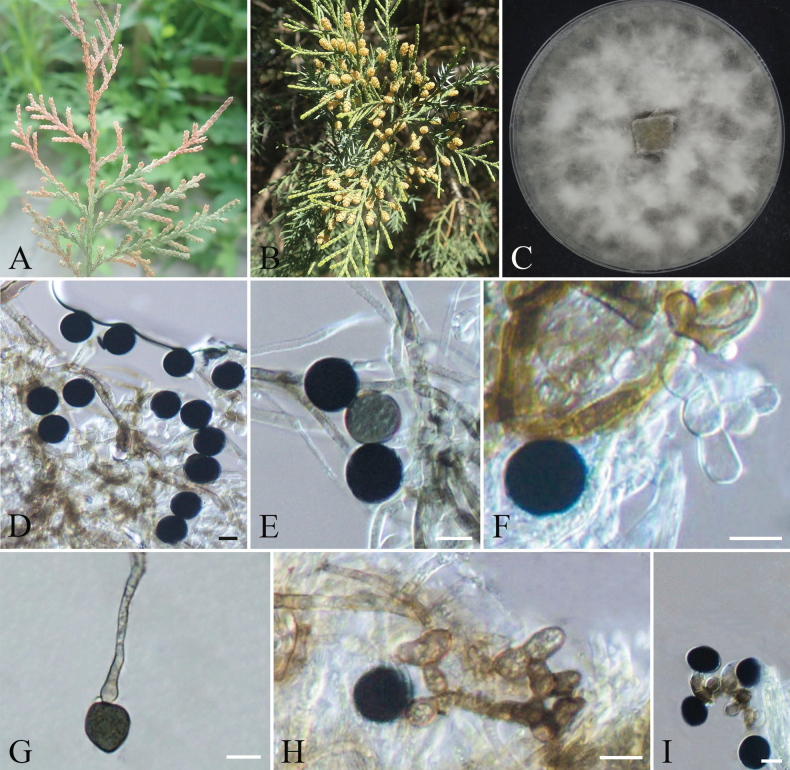
*Nigrospora philosophiae-doctoris* (CFCC 72628, CFCC 72637). A. Diseased scale leaves habit of *Platycladus orientalis*; B. Healthy strobili habit of *Juniperus chinensis*; C. Colony surface on PDA; D, E. Conidia; F–I. Conidiogenous cells. Scale bars: 10 µm (D–I).

#### ﻿Sordariomycetes O.E. Erikss. & Winka

**Sordariales Chadef. ex D. Hawksw. & O.E. Erikss**

##### Chaetomiaceae G. Winter

The family Chaetomiaceae, established by Winter (1885) with *Chaetomium* as the type genus (Wang et al. 2022), is characterized by membranous ascomatal walls, evanescent asci (dissolving upon maturation), unicellular ascospores, and a predominance of sexual morphs. Species delimitation within this family traditionally relies on morphological features of asci and ascospores, presence or absence of germ pores, types of ascomatal hairs, and structural details of ascomatal walls (Winter 1885; von Arx et al. 1984, 1986; Condé et al. 2023). Currently, Chaetomiaceae comprises 50 genera (Wang et al. 2022; Condé et al. 2023). Members of Chaetomiaceae exhibit remarkable phenotypic and ecological diversity, serving as critical resources in medical and economic contexts (Wang et al. 2022). They are globally distributed, predominantly as saprophytes, endophytes, or pathogens (Wang et al. 2022; AL-Rifaie and Ameen 2023; Condé et al. 2023). In this study, four Chaetomiaceae strains were isolated from *Platycladus orientalis* in the Ming Tombs area; they were identified as belonging to 3 genera and 3 species of fungi, namely *Achaetomium globosum*, *Arcopilus aureus*, and *Chaetomium globosum*.

###### ﻿ Achaetomium globosum

Taxon classificationFungiSordarialesChaetomiaceae

J.N. Rai & J.P. Tewari, Canad. J. Bot. 42(6): 693 (1964)

D368AD16-C135-5D81-B96B-469ACED27959

Fig. 17

####### Description.

**Sexual morph**: On CMA medium, sporulation initiated after approximately 30 days of cultivation. ***Ascomata*** Spherical to subellipsoidal, initially grayish-yellow, turning brownish to black at maturity, ostiolate, attached to the medium surface by aerial hyphae or sometimes partially embedded in the medium, 80–286 μm diam., peridium composed of textura intricata (interwoven hyphae), brownish in color. ***Asci*** Not observed. ***Ascospores*** Spherical to ellipsoidal, brownish, extruded in droplet form from ascomata, aseptate, unicellular, measuring 9.7–13.1 × 8.8–11.0 µm (av. ± S.D. = 11.6 ± 0.9 × 9.8 ± 0.6) μm in size. **Asexual morph**: Not observed during this study.

####### Culture characteristics.

Cultured on PDA medium for 7 days at 25 °C in the dark, the colony diameter can reach 60 mm. The aerial hyphae are flocculent, white at the initial stage and then turn light purple-pink. After 14 days, the colony color becomes purplish red and produces purple-pink pigments. It is not easy to sporulate on PDA medium. On CMA medium, the colony diameter reaches 60 mm after 7 days of culture. The colony is grayish yellow, and the aerial hyphae are light yellow. Sporulation begins on the surface of the medium after about 30 days of culture.

**Figure 17.**
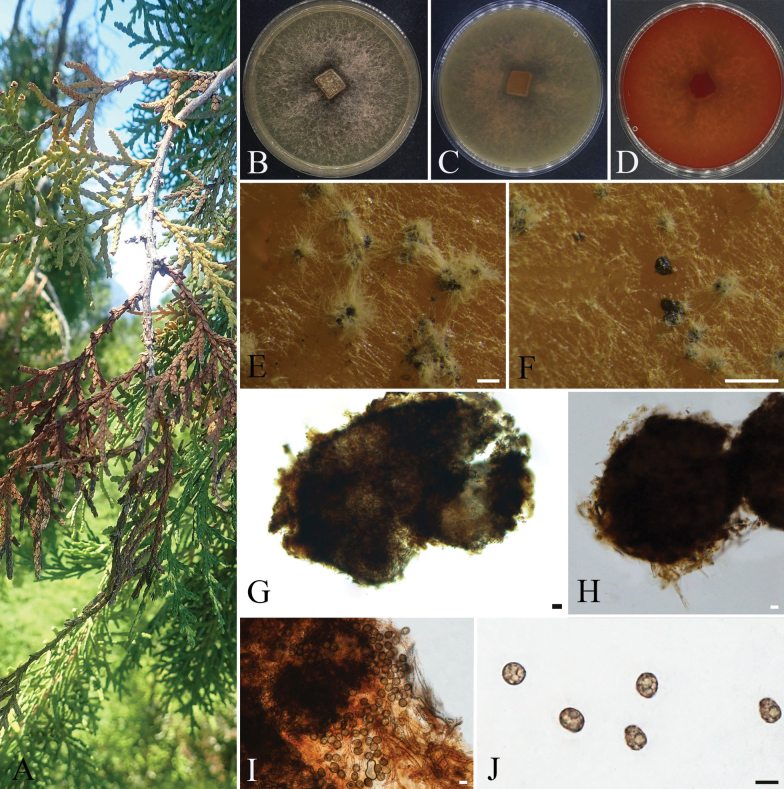
*Achaetomium globosum* (CFCC 72648). A. Diseased scale leaves habit of *Platycladus orientalis*; B, C. Colony surface and reverse on PDA for 7 days; D. The reverse side of the colony cultured on PDA for 14 days; E, F. Ascomata on CMA medium; G, H. Ascomata; I, J. Ascospores. Scale bars: 200 µm (E, F); 10 µm (G–J).

####### Specimens examined.

China • Beijing City, Changping District, Ming Tombs Reservoir, 40°14'48"N, 116°15'1"E, on the diseased scale leaves of *Platycladus orientalis*, 2 October 2024, Z.X. Bi, BJFC-S2565, living culture CFCC 72648.

####### Notes.

*Achaetomium globosum* was first isolated and described by Rai et al. (1964) from *Tamarindus indica*, with a subsequent record on *Parthenium* sp. (Pande 2008). Comprehensive phylogenetic and morphological analyses identified the fungal strain CFCC 72648 as *A. globosum*.

###### ﻿ Arcopilus aureus

Taxon classificationFungiSordarialesChaetomiaceae

(Chivers) X.Wei Wang & Samson, Studies in Mycology 84: 217 (2016)

CDFD4E87-5B5E-58E4-AD7F-10CABE1E0668

Fig. 18

####### Description.

**Sexual morph**: When cultured on PDA medium for approximately 30 days, sporulation begins. ***Ascomata*** subglobose to ovate, initially light brown, turning dark brown at maturity, superficial, 92–291 μm diam., and possess an ostiole. ***Ostiole*** tubular, dark brown, straight or curved, reaching up to 360 μm in length. ***Terminal hairs*** arcuate, with hooked and coiled apices, pale yellowish-brown, 107–341 μm in length. ***Asci*** fasciculate, clavate, evanescent, containing eight biseriately arranged ascospores, 15.0–30.4 × 7.6–12.3 µm (av. ± S.D. = 23.7 ± 4.2 × 9.8 ± 1.3). ***Ascospores*** unicellular, hyaline, and transparent when immature, becoming brown at maturity, fusiform, reniform, or limoniform, with 1–2 germ pores at each end, 6.9–10.3 × 4.3–6.1 µm (av. ± S.D. = 8.5 ± 0.6 × 5.3 ± 0.4) μm. **Asexual morph**: Not observed.

####### Cultural characteristics.

When cultured on PDA medium at 25 °C in darkness for 7 days, the colonies reached 55 mm in diameter, with abundant white aerial hyphae showing radial growth. After 10 days, the mycelium fully covered the Petri dish, forming concentric rings and continuing to expand outward; the colonies produced purple-red pigments that diffused throughout the agar surface. By 30 days, the colonies turned purple-black, and sporulating structures became visible on the medium surface.

**Figure 18.**
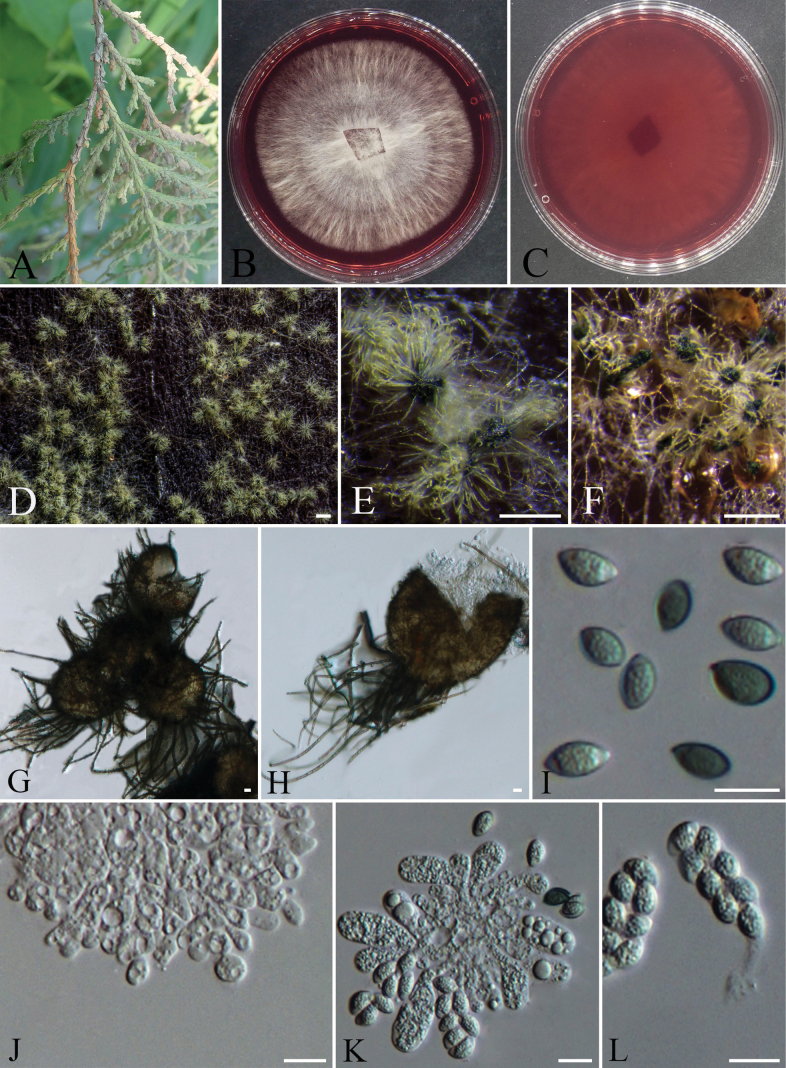
*Arcopilus aureus* (CFCC 72639). A. Diseased scale leaves habit of *Platycladus orientalis*; B, C. Colony surface and reverse on PDA; D–F. Ascomata on PDA medium; G, H. Ascomata; I. Ascospores; J. The basal cell of the ascus; K, L. Ascus and ascospores. Scale bars: 200 µm (D–F); 10 µm (G–L).

####### Specimens examined.

China • Beijing City, Changping District, Ming Tombs Reservoir, “40°14'57"N, 116°15'54"E”, on the diseased scale leaves of *Platycladus orientalis*, 23 February 2025, Z.X. Bi, BJFC-S2571, living culture CFCC 72639.

####### Notes.

The genus *Arcopilus* was introduced by Wang et al. (2016), with *Arcopilus aureus* designated as the type species. This genus is characterized by colonies producing yellow to orange or red to rust-colored pigments, arcuate perithecial hairs, and ascospores with diverse morphologies (Wang et al. 2016). *A. aureus* is an endophyte widely associated with various plants (Zimowska and Nicoletti 2023) and also acts as a pathogenic fungus. Reported infections caused by *A. aureus* include leaf black spot disease in *Pseudostellaria heterophylla* (Yuan et al. 2021), leaf spot disease in *Cucumis melo* (Wei et al. 2024), and gray spot disease in tobacco (Yang et al. 2024). Comprehensive phylogenetic and morphological analyses identified the fungal strain CFCC 72639 as *A. aureus*.

###### ﻿ Chaetomium globosum

Taxon classificationFungiSordarialesChaetomiaceae

Kunze, Mykol. Hefte 1: 16 (1817).

B7A09D1D-A133-5A8D-9EA3-36C2FF473923

Fig. 19

####### Description.

**Sexual morph: *Ascomata*** densely distributed on the surface of PDA medium, initially pale yellow, maturing to yellowish-black after 2 weeks, superficial on the medium, globose to ovate, with an apical ostiole, 158–269 × 136–186 µm, surrounded by ascomatal hairs, the ascomatal wall is brownish and composed of textura intricate. ***Terminal hairs*** initially pale yellow, turning brownish-yellow with age, base dark brown, apex pale yellowish-brown, sinuous, septate, unbranched, 146–468 µm long, 1.7–3.9 µm wide at the base. ***Asci*** fasciculate, clavate, stipitate, hyaline, 8-spored, evanescent, 25.6–47.2 × 10.3–17.9 µm (av. ± S.D. = 37.5 ± 5.4 × 14.1 ± 2.1). ***Ascospores*** ovoid, hyaline when immature, becoming brown at maturity, 8.5–10.7 × 6.4–8.5 µm (av. ± S.D. = 9.6 ± 0.5 × 7.5 ± 0.5). **Asexual morph**: Not observed.

####### Cultural characteristics.

Initially, colonies on PDA medium appeared white. After approximately 7 days, they turned pale yellow and began producing golden-brown ascomata from the center. Within 10 days, the ascomata densely covered the entire medium surface. By 14 days, pale orange-yellow exudates were observed. Upon maturation, ascospores were released through the apical ostioles.

**Figure 19.**
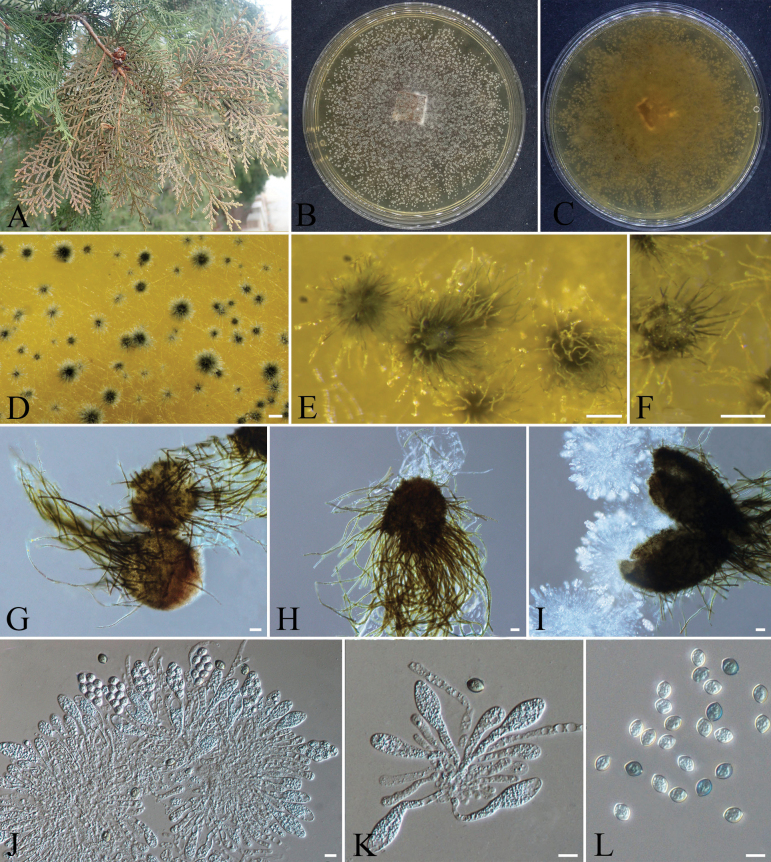
*Chaetomium globosum* (CFCC 72642). A. Diseased scale leaves habit of *Platycladus orientalis*; B, C. Colony surface and reverse on PDA; D–F. Ascomata on PDA medium; G–I. Ascomata; J, K. Asci; L. Ascospores. Scale bars: 200 µm (D–F); 10 µm (G–L).

####### Specimens examined.

China • Beijing City, Changping District, Ming Tombs Reservoir, 40°14'47"N, 116°15'54"E”, on the diseased scale leaves of *Platycladus orientalis*, 23 February 2025, Z.X. Bi, BJFC-S2572, living cultures CFCC 72642; China • Beijing City, Changping District, Mangshan National Forest Park, Ming Tombs, 40°15'36"N, 116°16'40"E, on the diseased scale leaves of *P. orientalis*, 23 November 2024, Z.X. Bi &W.K. Gao, BJFC-S2573, living culture CFCC 72645.

####### Notes.

*Chaetomium* was introduced by Kunze, with *Chaetomium globosum* designated as the type species (Kunze and Schmidt 1817). *C. globosum* is a widely distributed endophytic fungus, recorded on numerous plants including *Actinidia chinensis*, *Artemisia argyi*, *Descurainia sophia*, *Glycine max*, *Juncus* sp., *Oryza sativa*, *Platycladus orientalis*, and *Solanum lycopersicum* (Guo 2012; Wang et al. 2016). As a significant resource fungus, it exhibits critical biological functions such as antimicrobial activity, biocontrol potential, and plant growth promotion (Ye et al. 2013; Zhao et al. 2017; Tian et al. 2022). In this study, two fungal strains isolated from diseased scale leaves of *P. orientalis* were analyzed. Based on comprehensive phylogenetic and morphological analyses, strains CFCC 72642 and CFCC 72645 were identified as *C. globosum*.

#### ﻿Sordariomycetes O.E. Erikss. & Winka

**Xylariales Nannf**

**Sporocadaceae Corda**

##### *Seiridium* Nees

The genus *Seiridium* was introduced by Nees (1817), with *Seiridium marginatum* designated as the type species (Bonthond et al. 2018; Li et al. 2022). *Seiridium* species primarily exist as phytopathogens, widely distributed globally and causing significant economic losses, particularly through infections of Cupressaceae plants. *Seiridium cardinale*, *S. cupressi*, and *S. unicorne* are recognized as the most dangerous parasitic fungi for Cupressaceae, identified as the primary pathogens responsible for cypress canker pandemics (Boesewinkel 1983; Graniti 1986, 1998; Bonthond et al. 2018). In this study, one fungal strain isolated from cankered twigs of *Platycladus orientalis* in the Ming Tombs area was identified as *S. unicorne*.

###### ﻿ Seiridium unicorne

Taxon classificationFungiXylarialesSporocadaceae

(Cooke & Ellis) B. Sutton, Mycol. Pap. 138: 74 (1975)

390F9897-EEC0-5836-95FD-701F6CE3D4FC

Fig. 20

####### Description.

**Sexual morph**: Not observed. **Asexual morph: *Fruiting bodies*** scattered on the surface of *Platycladus orientalis* branches, carbon-black to jet-black; ***Conidiomata*** acervular, immersed to erumpent through bark tissue, black, subglobose, scattered, unilocular; wall brownish, 65–255 µm diam. ***Conidiophores*** long-cylindrical, hyaline, thin-walled, septate, occasionally branched, 16.3–51.4 × 1.0–2.6 µm; ***Conidiogenous cells*** hyaline, thin-walled, smooth, cylindrical, solitary, 5.9–17.5 × 1.1–4.2 µm (av. ± S.D. = 10.8 ± 3.2 × 2.0 ± 0.6). ***Conidia*** falcate to lunate, hyaline when immature, becoming pale brown to yellowish-brown at maturity, 5-septate, curved, with one hyaline apical appendage and one basal appendage, total conidial dimensions 19.4–29.8 × 6.2–11.9 µm (av. ± S.D. = 24.5 ± 0.4 × 9.6 ± 1.2), basal cell obconical, hyaline to pale brown, truncate, 2.5–7.1 µm long, the first cell from the basal cell upwards is 3.5–7.1 µm long, the second cell is 3.2–6.7 µm long, the third cell 3.1–6.0 µm long, the fourth cell 3.6–6.9 µm long, the apical cell conical, smooth, and hyaline, with a length of 1.6–5.7 µm. ***Appendages*** cylindrical, the apical appendages are mostly centric, 4.0–8.9 µm long, the basal appendages are mostly eccentric, 4.0–8.6 µm long.

**Figure 20.**
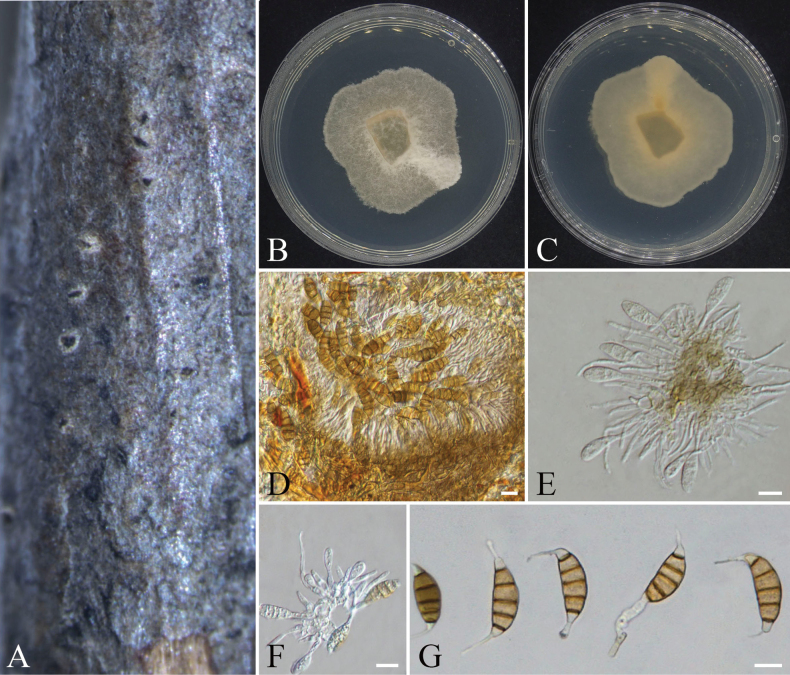
*Seiridium unicorne* (CFCC 72631). A. Conidiomata on a diseased branch habit of *Platycladus orientalis*; B, C. Colony surface and reverse on PDA; D. Conidiomata and conidia; E, F. Conidiogenous cells; G. Conidia. Scale bars: 10 µm (D–G).

####### Cultural characteristics.

On PDA medium, colonies exhibited appressed growth with a sparse, felt-like texture and slow expansion rates, reaching approximately 30 mm in diameter after 7 days of incubation. Aerial mycelium was poorly developed and diffuse. After 2 weeks, a pale yellow pigmentation became visible on the colony reverse.

####### Specimens examined.

China • Beijing City, Changping District, Ming Tombs Longshan Sub-farm, 40°14'21"N, 116°13'15"E, on the dead branches of *Platycladus orientalis*, 18 July 2024, Z.X. Bi & C.M. Tian. BJFC-S2581, living culture CFCC 72631.

####### Notes.

The genus *Seiridium* can be distinguished from other genera by its conidia with five septa (Li et al. 2022). *Seiridium unicorne* has been documented to infect hosts across diverse plant families, including Anacardiaceae, Caprifoliaceae, Cornaceae, Cupressaceae, Hamamelidaceae, Rosaceae, and Vitaceae (Guba 1961; Boesewinkel 1983; Cho and Shin 2004; Bonthond et al. 2018). Phylogenetic analysis revealed that the studied strains cluster within the same clade as reference strains of *S. unicorne* with a high support value of 100/1 (ML/BI) (Fig. 7). In terms of morphology, the maximum lengths of the basal cells (2.5–7.1 µm vs. 3–5.5 μm) and the first cell counted upwards from the basal cell (3.5–7.1 µm vs. 3.5–5.5 μm) in the conidia of the strains in this study are slightly larger than those of the reference species *S. unicorne* (Bonthond et al. 2018). However, the differences are not significant, and the remaining morphological characteristics are basically consistent with the previous descriptions of this species. Therefore, based on the above evidence, we identified this strain as *S. unicorne*.

## ﻿Discussion

This study isolated 22 fungal strains from diseased leaves and twigs, as well as healthy strobili and mature cones of cypress (*Juniperus chinensis*, *J. procumbens*, and *Platycladus orientalis*) in the Ming Tombs area of Beijing. Identification revealed that these isolates belong to 13 species across 8 fungal genera, including *Achaetomium globosum*, *Aplosporella hesperidica*, *A. javeedii*, *A. prunicola*, *Arcopilus aureus*, *Chaetomium globosum*, *Neofusicoccum occulatum*, *Nigrospora oryzae*, *N. osmanthi*, *N. philosophiae-doctoris*, *N. platycladiensis*, *Seiridium unicorne*, and *Spegazzinia juniperi*. Among these, *N. platycladiensis* and *S. juniperi* are described as novel species. *A. hesperidica* was recorded for the first time on *P. orientalis*. *N. philosophiae-doctoris* represents the first record on both *J. chinensis* and *P. orientalis*. In this study, a total of 12 fungal species were isolated from *P. orientalis*, 3 species were obtained from *J. chinensis*, and 1 was isolated from *J. procumbens*. Furthermore, 12 ascomycete species were isolated from diseased cypress leaves and branches, whereas 4 fungal species were obtained from healthy tissues.

*Aplosporella* is primarily characterized by the formation of multilocular pycnidia (multi-chambered fruiting bodies), producing brown, aseptate, verruculose conidia, and the presence of filiform paraphyses (Sutton 1980; Damm et al. 2007). In this study, 3 *Aplosporella* species were isolated from *P. orientalis*, namely *A. hesperidica*, *A. javeedii*, and *A. prunicola*. *A. javeedii* was found not only in withered branches but also within healthy strobili tissues of the host. According to the literature, *A. javeedii* exhibits the broadest host range within the genus, having been reported on hosts spanning more than 10 plant families (Fan et al. 2015; Zhu et al. 2018; Pan et al. 2019; Lin et al. 2023b; Wu et al. 2024). Significantly, although specimens from 3 different cypress species were collected, *Aplosporella* isolates were obtained exclusively from *P. orientalis*. This phenomenon warrants further investigation and provides new research directions for exploring the distribution patterns of this fungal genus within cypress hosts.

This study isolated 3 species belonging to the family Chaetomiaceae from diseased scale-like leaves of *P. orientalis*: *Arcopilus aureus*, *Achaetomium globosum*, and *Chaetomium globosum*. Chaetomiaceae species exhibit remarkable phenotypic and ecological diversity and hold significant value in medical and economic contexts, representing important resource fungi (Wang et al. 2022). The family is distributed globally, existing primarily as saprophytes, endophytes, and pathogens in natural environments (Wang et al. 2022; Al-Rifaie and Ameen 2023; Condé et al. 2023). Studies indicate that *A. aureus* has a wide geographical distribution. It frequently exists as an endophyte in symbiotic relationships with host plants, demonstrating high adaptability to its ecological niche (Zimowska and Nicoletti 2023). Reports also suggest that this fungus possesses potential pathogenicity, capable of causing black spot, leaf spot, and grey spot diseases in plants (Yuan et al. 2021; Wei et al. 2024; Yang et al. 2024). In this study, *A. aureus*, *A. globosum*, and *C. globosum* were isolated from diseased scale-like leaves of *P. orientalis*. Whether these fungi can induce disease necessitates experimental confirmation through subsequent pathogenicity assays.

*Nigrospora* is not only an endophyte widely present in various host plants but also a potential pathogen on many plants in different regions (Wang et al. 2016; Liu et al. 2024). In this study, 3 previously reported species of this genus (*N. oryzae*, *N. osmanthi*, and *N. philosophiae-doctoris*), as well as one new species, *Nigrospora platycladiensis*, were isolated from cypress. Among these, *N. osmanthi* and *N. philosophiae-doctoris* were isolated from both diseased leaves and healthy strobili of cypress. *N. oryzae* and *N. platycladiensis* were isolated from diseased leaves. Notably, *N. oryzae* was isolated from diseased parts of both *P. orientalis* and *J. procumbens*, indicating its potential broad host range. Early taxonomic studies of *Nigrospora* primarily relied on morphological characteristics for species delimitation (Mason 1927, 1933; Wang et al. 2017). However, research by Wang et al. (2017) demonstrated that although some species within *Nigrospora* exhibit extremely similar morphology, they belong to distinct phylogenetic clades, often showing overlapping conidial size ranges. Although the newly described species *N. platycladiensis* sp. nov. exhibits partially overlapping morphological characteristics with its closely related species *N. guangdongensis*, the two species show clear distinctions in their geographical distribution and host origins: *N. platycladiensis* was isolated from *P. orientalis* in Beijing, China, whereas *N. guangdongensis* was collected from *Cunninghamia lanceolata* in Hebei, China (Tian et al. 2020). Therefore, the identification of *Nigrospora* cannot rely solely on morphological features and requires an integrated approach combining both morphological and phylogenetic analyses for proper species classification and delimitation (Wang et al. 2017).

*Neofusicoccum occulatum* and *Seiridium unicorne*, identified as pathogens causing twig blight and canker diseases in cypress trees, have been confirmed to be closely associated with cypress diseases (Liu et al. 2022; Guo 2023). In this study, both of these fungal species were isolated from withered branches of *P. orientalis*, providing new corroborating evidence for previous research findings. *N. occulatum* is known to infect various Cupressaceae plants and has been reported on species such as *Chamaecyparis lawsoniana*, *Cupressus funebris*, *Juniperus communis*, and *Thujopsis dolabrata* (Zlatkovic 2016; Li et al. 2022). *S. cardinale*, *S. cupressi*, and *S. unicorne* are considered the most dangerous parasitic fungi for Cupressaceae plants (Bonthond et al. 2018). However, only *S. unicorne* was isolated in this study. This discrepancy might be attributed to factors such as host plant species, geographical location, and the scale of sampling. According to Guo (2023), reports on *Seiridium* fungi in China are relatively scarce. To date, no studies have been identified that investigate the presence of *Seiridium* on the branches and leaves of Cupressaceae plants in the Ming Tombs area of Beijing.

Species of *Spegazzinia* exhibit an extremely wide geographical distribution across various ecosystems (Dai et al. 2025). They primarily exist as endophytes within host organisms or as saprobes on decaying plant debris (Hashemlou et al. 2023; Dai et al. 2025). *Spegazzinia* has been recorded on multiple host plants, including *Brachypodium* sp., *Musa* sp., *Radermachera sinica*, and *Saccharum officinarum*, and has also been reported in air samples (Mena-Portales et al. 2017; Jayasiri et al. 2019; Samarakoon et al. 2020; Hashemlou et al. 2023; Dai et al. 2025). In this study, a new species of this genus, *Spegazzinia juniperi*, was isolated from healthy cones of *J. chinensis*. Phylogenetically, *S. juniperi* formed a distinct branch (Fig. 3). Morphologically, it can be distinguished from other species by its granular, slightly moist sporodochia and characteristic conidial dimensions.

A preliminary investigation into the diversity of ascomycetes on cypress trees in the Ming Tombs area of Beijing revealed that the ascomycete species in this region possess a certain degree of richness. However, the number of specimens collected in this study is limited. In the future, it will be necessary to expand the scale of specimen collection to more fully verify the existing research results and explore additional ascomycete groups on cypress.

## Supplementary Material

XML Treatment for Spegazzinia juniperi

XML Treatment for Nigrospora platycladiensis

XML Treatment for Aplosporella hesperidica

XML Treatment for Aplosporella javeedii

XML Treatment for Aplosporella prunicola

XML Treatment for Neofusicoccum occulatum

XML Treatment for Nigrospora oryzae

XML Treatment for Nigrospora osmanthi

XML Treatment for Nigrospora philosophiae-doctoris

XML Treatment for Achaetomium globosum

XML Treatment for Arcopilus aureus

XML Treatment for Chaetomium globosum

XML Treatment for Seiridium unicorne

## ﻿Additional information

### Use of AI

No use of AI was reported.

### Author ORCIDs

Zixian Bi https://orcid.org/0009-0008-1186-8995

Yingying Wu https://orcid.org/0009-0007-5095-2738

Chengming Tian https://orcid.org/0000-0002-3352-7664

### Data availability

All of the data that support the findings of this study are available in the main text or Supplementary Information.

## Supplementary materials

Supplementary material 1 Fungal species isolated from different tissues of host plants Data type xlsx https://binary.pensoft.net/file/1441410This dataset is made available under the Open Database License (http://opendatacommons.org/licenses/odbl/1.0/). The Open Database License (ODbL) is a license agreement intended to allow users to freely share, modify, and use this Dataset while maintaining this same freedom for others, provided that the original source and author(s) are credited.Zixian Bi, Yingying Wu, Shuji Li, Chengming Tian

Supplementary material 2 The primer sequences (forward and reverse) and PCR conditions for each genus Data type xlsx Explanation note Suppl. material 2 contains the sequence information of each genus involved in this study for phylogenetic analysis, as well as the primer sequences (forward and reverse) and PCR conditions corresponding to each genus. https://binary.pensoft.net/file/1441411This dataset is made available under the Open Database License (http://opendatacommons.org/licenses/odbl/1.0/). The Open Database License (ODbL) is a license agreement intended to allow users to freely share, modify, and use this Dataset while maintaining this same freedom for others, provided that the original source and author(s) are credited.Zixian Bi, Yingying Wu, Shuji Li, Chengming Tian

Supplementary material 3 Phylogenetic trees of Didymosphaeriaceae and *Neofusicoccum* Data type rar Explanation note **figure S1.**ML phylogenetic tree of Didymosphaeriaceae based on combined ITS, LSU, SSU, and *tef1-α* sequence data; **figure S2.**ML phylogenetic tree of *Neofusicoccum* isolates based on combined ITS, *tef1-α*, *tub2*, and *rpb2* sequence data. https://binary.pensoft.net/file/1441412This dataset is made available under the Open Database License (http://opendatacommons.org/licenses/odbl/1.0/). The Open Database License (ODbL) is a license agreement intended to allow users to freely share, modify, and use this Dataset while maintaining this same freedom for others, provided that the original source and author(s) are credited.Zixian Bi, Yingying Wu, Shuji Li, Chengming Tian
